# A large multi-ethnic genome-wide association study identifies novel genetic loci for intraocular pressure

**DOI:** 10.1038/s41467-017-01913-6

**Published:** 2017-12-13

**Authors:** Hélène Choquet, Khanh K. Thai, Jie Yin, Thomas J. Hoffmann, Mark N. Kvale, Yambazi Banda, Catherine Schaefer, Neil Risch, K. Saidas Nair, Ronald Melles, Eric Jorgenson

**Affiliations:** 1 Kaiser Permanente Northern California (KPNC), Division of Research, Oakland, CA 94612 USA; 2 Institute for Human Genetics, University of California San Francisco (UCSF), San Francisco, CA 94143 USA; 3 Department of Epidemiology and Biostatistics, UCSF, San Francisco, CA 94158 USA; 4 Departments of Ophthalmology and Anatomy, School of Medicine, UCSF, San Francisco, CA 94143 USA; 5 KPNC, Department of Ophthalmology, Redwood City, CA 94063 USA

## Abstract

Elevated intraocular pressure (IOP) is a major risk factor for glaucoma, a leading cause of blindness. IOP heritability has been estimated to up to 67%, and to date only 11 IOP loci have been reported, accounting for 1.5% of IOP variability. Here, we conduct a genome-wide association study of IOP in 69,756 untreated individuals of European, Latino, Asian, and African ancestry. Multiple longitudinal IOP measurements were collected through electronic health records and, in total, 356,987 measurements were included. We identify 47 genome-wide significant IOP-associated loci (*P* < 5 × 10^−8^); of the 40 novel loci, 14 replicate at Bonferroni significance in an external genome-wide association study analysis of 37,930 individuals of European and Asian descent. We further examine their effect on the risk of glaucoma within our discovery sample. Using longitudinal IOP measurements from electronic health records improves our power to identify new variants, which together explain 3.7% of IOP variation.

## Abstract (web summary)

Intraocular pressure (IOP) is a major risk factor for glaucoma. Here, Choquet and co-authors perform a multi-ethnic genome-wide association study of repeat IOP measurements in 69,756 individuals and identify 40 novel loci, 36 of which show directionally consistent effects in glaucoma.

## Introduction

Elevated intraocular pressure (IOP) is the main modifiable risk factor in glaucoma development and progression^1, 2^, a leading cause of blindness worldwide^3^. IOP is controlled by balancing aqueous humor production by the ciliary body with drainage through the structures located at the junction where the cornea meets the iris^4^. Inefficient aqueous humor drainage leads to elevated IOP, which, in turn, contributes to glaucomatous neurodegeneration characterized by progressive loss of retinal ganglion cells and corresponding visual field damage. The mechanisms controlling aqueous humor dynamics and IOP regulation are still poorly understood. Gene identification may help uncover the mechanisms underlying IOP variation, and, consequently, glaucoma susceptibility.

Twin and family studies indicate that IOP has a moderate genetic component, with heritability estimates ranging from 0.29 to 0.67^5^. Genetic association studies have reported 11 loci associated with IOP^6–13^, accounting for up to 1.5% of the variance for this ocular trait^8^. The limited proportion of variance explained by known loci suggests that additional loci remain to be discovered.

IOP can vary owing to different factors, including time of day at which the measurement was taken, corneal thickness, medications, different measurement techniques that were employed (i.e., Goldmann applanation tonometer, Tono-Pen XL, or others), and demographic factors such as age and racial/ethnic background^14–16^. These sources of variability may reduce the power to detect IOP loci in studies that rely on a single, cross-sectional measurement for each study participant. Incorporating information from multiple longitudinal measurements collected through electronic health records has been shown to improve the precision of the characterization of phenotypes by reducing measurement error (such as for blood pressure), and, in turn, improve the power to detect novel associations with genetic loci^17^.

Here, we undertake a GWAS of IOP in the large and ethnically diverse Genetic Epidemiology Research in Adult Health and Aging (GERA) cohort, including 356,987 IOP measurements from 69,756 individuals (not treated with IOP lowering medication), the largest sample to date. We then validate genome-wide significant associations using summary statistics from an external independent GWAS meta-analysis of 37,930 individuals of European and Asian descent^11^. We further examine their effect on the risk of glaucoma within our GERA discovery sample. Through the increase in sample size in the current study and the use of multiple IOP measurements, we reason that our study would identify a substantial number of novel genetic loci associated with IOP. A better understanding of the genetic factors underlying IOP can provide insight into the risk factors that underlie glaucoma susceptibility and progression.

## Results

### GERA cohort

**Fig. 1:**
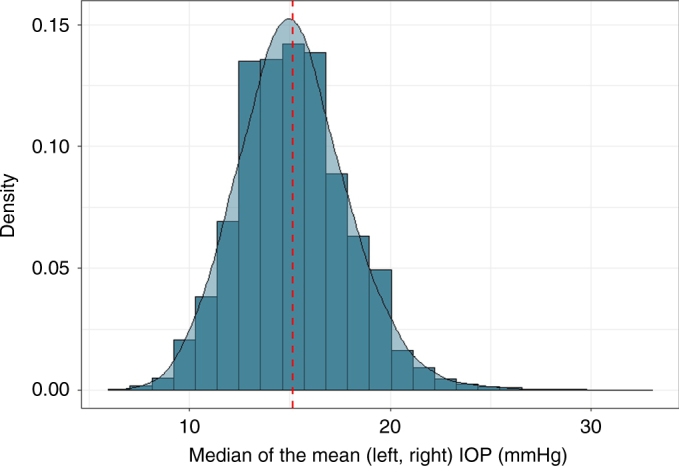
Distribution of the median of the mean (left, right) of IOP in GERA cohort subjects. A total of 356,987 IOP measurements from 69,756 individuals were used to create this chart showing the normal distribution of the median of the IOP mean from both eyes

We conducted the primary discovery analysis in 69,756 individuals from four race/ethnicity groups (non-Hispanic whites, 81.5%; Hispanic/Latinos, 8.2%; East Asians, 7.3%; and African-Americans, 3.0%) in the GERA cohort (Table 1). The GERA cohort is part of the Kaiser Permanente Research Program on Genes, Environment, and Health (RPGEH), an unselected cohort of adult participants who are members of an integrated health care delivery system (Kaiser Permanente Northern California), with ongoing longitudinal records from vision examinations. The 69,756 untreated GERA subjects had a total of 356,987 IOP measurements (an average of 5.8 IOP measurements per individual). In this analysis, we used the mean IOP of both eyes and selected the median of the mean IOP of both eyes (Fig. 1 and Table 1). On average, IOP levels were slightly higher in females than in males across the race/ethnicity groups, and East Asian individuals had lower levels of IOP compared to the other groups.

**Table 1:** Characteristics of GERA subjects with intraocular pressure measurements by sex, and race/ethnicity group

		Participants *N* (%)	Median IOP (od,os) (mmHg) mean ± SD	Age at median IOP (years) mean ± SD	*N* IOP Meas. mean ± SD
All		69,756 (100.0)	15.11 ± 2.71	68.36 ± 12.53	5.75 ± 6.76
Sex	Female	41,958 (60.1)	15.25 ± 2.65	67.00 ± 13.04	5.80 ± 6.72
	Male	27,798 (39.9)	14.90 ± 2.78	70.42 ± 11.41	5.67 ± 6.81
Race/ethnicity	NHW	56,819 (81.5)	15.16 ± 2.72	69.33 ± 12.06	5.81 ± 6.93
	H/L	5,748 (8.2)	15.22 ± 2.67	63.98 ± 13.85	5.33 ± 5.90
	EAS	5,119 (7.3)	14.34 ± 2.54	63.65 ± 13.65	5.63 ± 5.83
	AFR	2,070 (3.0)	15.31 ± 2.81	65.56 ± 12.79	5.62 ± 6.16
Glaucoma	Cases	2,338	15.85 ± 2.90	75.07 ± 9.43	17.49 ± 11.21
	Controls	58,172	14.91 ± 2.61	67.80 ± 12.78	4.83 ± 5.72

*N*, number; SD, standard deviation; od, right eye; os, left eye; meas, measurements; NHW, non-Hispanic whites; H/L, Hispanic/Latinos; EAS, East Asians; AA, African-Americans

### Novel IOP loci in GERA

**Fig. 2:**
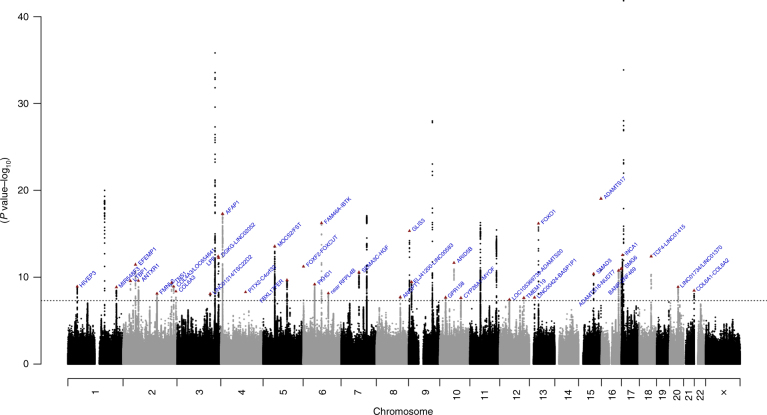
Manhattan plot of the GERA discovery cohort trans-ethnic GWAS meta-analysis of IOP. GWAS of IOP was conducted in 69,756 individuals from four race/ethnicity groups (non-Hispanic white, Latino, East Asian, and African American). Association results (–log_10_ *P* values) are plotted for each chromosome. The locus/gene names are indicated for each novel locus associated with IOP, and the lead SNP at each IOP novel loci is represented by a red triangle

In our discovery GWAS analysis, we identified 47 independent genome-wide significant (*P* < 5 × 10^–8^) loci associated with IOP in the trans-ethnic meta-analysis; the genomic inflation factor, *λ*, of 1.12, is reasonable for a sample of this size (Fig. 2, Table 2 and Supplementary Fig. 1a). Of the 47 identified loci, 40 were novel (85.1%), that is, not previously reported to be associated with IOP. These 47 associations with IOP were also examined in each individual race/ethnicity group (Supplementary Table 1 and Supplementary Fig. 1b–e).

**Table 2:** Lead genome-wide significant SNP for each independent locus identified in the GERA discovery GWAS of IOP

SNP	Chr	Pos	Locus	Alleles	GERA discovery cohort		External replication cohort		Discover+replication meta-analysis	
					*β*	*P* value	*β*	*P* value	*β*	*P* value
rs1866758	1	42318868	*HIVEP3*	T/C	0.10	1.2 × 10^−9^	0.07	0.0063	0.09	4.6 × 10^−11^
rs6668108	1	165691320	*LOC440700-TMCO1*	A/G	0.22	1.0 × 10^−20^	0.24	8.2 × 10^−11^	0.23	6.8 × 10^−30^
rs596169	1	219147419	*MIR548F3*	A/G	−0.18	1.4 × 10^−9^	−0.18	0.0006	−0.18	3.5 × 10^−12^
rs115179432	2	33348679	*LTBP1*	A/G	−0.19	2.7 × 10^−10^	−0.10	0.07	−0.17	1.3 × 10^−10^
rs7426380	2	56095010	*EFEMP1*	A/G	−0.11	3.5 × 10^−12^	−0.07	0.0059	−0.10	2.1 × 10^−13^
rs6732795	2	69411517	*ANTXR1*	A/C	−0.10	2.6 × 10^−10^	−0.09	0.0002	−0.10	2.0 × 10^−13^
rs55692468	2	153361375	*FMNL2*	T/G	−0.09	7.7 × 10^−9^	−0.10	1.3 × 10^−5^	−0.09	5.4 × 10^−13^
rs1035673	2	218675533	*TNS1*	T/C	−0.10	5.0 × 10^−10^	−0.07	0.0023	−0.09	6.9 × 10^−12^
rs143937055	2	228143966	*COL4A3/LOC654841*	TTTG/T	0.10	1.2 × 10^−9^	0.10	0.0002	0.10	9.0 × 10^−13^
rs7599762	2	238322885	*COL6A3*	G/C	−0.13	4.3 × 10^−9^	−0.05	0.15	−0.10	1.2 × 10^−8^
rs11710845	3	150065280	*LINC01214/TSC22D2*	C/T	0.10	8.2 × 10^−9^	0.05	0.08	0.09	6.9 × 10^−9^
rs7635832	3	171989276	*FNDC3B*	T/G	0.24	1.5 × 10^−36^	0.22	6.6 × 10^−13^	0.23	9.3 × 10^−48^
rs9853115	3	186131600	*DGKG-LINC02052*	T/A	0.11	4.1 × 10^−13^	0.08	0.0009	0.10	2.5 × 10^−15^
rs13076750	3	188059443	*LPP*	A/G	0.12	6.3 × 10^−13^	0.06	0.0118	0.10	1.3 × 10^−13^
rs28795989	4	7891545	*AFAP1*	A/G	0.14	4.9 × 10^−18^	0.10	0.0001	0.13	6.0 × 10^−21^
rs17527016	4	111963719	*PITX2-C4orf32*	C/T	0.11	5.4 × 10^−9^	0.09	0.0137	0.10	2.6 × 10^−10^
rs4865762	5	52582931	*MOCS2/FST*	C/A	0.12	2.8 × 10^−14^	0.08	0.0006	0.11	1.4 × 10^−16^
rs73220188	5	108053612	*FBXL17/FER*	T/C	0.15	2.2 × 10^−10^	0.14	0.0001	0.15	1.4 × 10^−13^
rs2745572	6	1548369	*FOXF2-FOXCUT*	A/G	0.11	5.9 × 10^−12^	0.12	1.3 × 10^−6^	0.11	4.4 × 10^−17^
rs1396046	6	51536992	*PKHD1*	G/A	−0.10	6.6 × 10^−10^	−0.04	0.07	−0.08	7.0 × 10^−10^
rs2875087	6	82616083	*FAM46A-IBTK*	C/T	0.13	6.0 × 10^−17^	0.07	0.0037	0.11	9.0 × 10^−18^
rs141917145	6	113375846	*near RFPL4B*	AAAGAAACAAG/A	0.10	7.2 × 10^−9^	0.04	0.13	0.08	1.2 × 10^−8^
rs1509922	7	80858944	*SEMA3C-HGF*	G/A	0.10	2.7 × 10^−11^	0.06	0.0197	0.09	5.7 × 10^−12^
rs6968419	7	115823384	*TFEC-TES*	A/G	0.13	8.3 × 10^−18^	0.09	0.0001	0.12	1.8 × 10^−20^
rs10105844	8	108290872	*ANGPT1*	G/A	−0.10	2.0 × 10^−8^	−0.08	0.0025	−0.09	2.2 × 10^−10^
rs2224492	9	4237546	*GLIS3*	A/G	−0.13	4.9 × 10^−16^	−0.06	0.0117	−0.11	3.8 × 10^−16^
rs1831902	9	13558317	*FLJ41200-LINC00583*	C/T	−0.12	3.6 × 10^−10^	−0.07	0.0160	−0.10	5.3 × 10^−11^
rs2472493	9	107695848	*AK311445/ABCA1*	G/A	0.17	1.1 × 10^−28^	0.17	2.3 × 10^−13^	0.17	1.8 × 10^−40^
rs11014632	10	25877651	*GPR158*	A/G	0.08	2.2 × 10^−8^	0.03	0.18	0.07	5.3 × 10^−8^
rs5785510	10	63837289	*ARID5B*	CA/C	0.11	2.2 × 10^−12^	0.10	9.7 × 10^−5^	0.11	1.0 × 10^−15^
rs66479974	10	95049397	*CYP26A1-MYOF*	CAG/C	0.09	2.4 × 10^−8^	0.09	0.0005	0.09	4.8 × 10^−11^
rs79390637	11	47456867	*PSMC3/RAPSN*	C/A	0.14	5.3 × 10^−17^	0.10	0.0003	0.13	1.4 × 10^−19^
rs199800298	11	120348583	*ARHGEF12*	AG/A	−0.13	3.6 × 10^−16^	−0.13	5.9 × 10^−7^	−0.13	1.2 × 10^−21^
rs7977237	12	43700341	*LOC105369739-ADAMTS20*	T/A	0.09	3.8 × 10^−8^	0.04	0.087	0.08	2.8 × 10^−8^
rs74481774	12	108987230	*TMEM119*	A/G	0.31	2.4 × 10^−8^	0.08	0.41	0.25	1.5 × 10^−7^
rs9552680	13	23235285	*LINC00424-BASP1P1*	C/T	−0.09	2.2 × 10^−8^	−0.06	0.0113	−0.08	1.2 × 10^−9^
rs11616662	13	41119466	*FOXO1*	G/A	0.21	6.6 × 10^−17^	0.12	0.0056	0.19	8.1 × 10^−18^
rs12912045	15	67467297	*SMAD3*	C/T	−0.12	4.0 × 10^−11^	−0.14	2.3 × 10^−7^	−0.12	6.2 × 10^−17^
rs72755233	15	100692953	*ADAMTS17*	G/A	−0.24	9.0 × 10^−20^	−0.19	0.0023	−0.23	1.2 × 10^−21^
rs75828804	16	77591935	*ADAMTS18-NUDT7*	G/A	−0.18	1.7 × 10^−11^	−0.19	0.0004	−0.18	2.9 × 10^−14^
rs12926024	16	88331309	*BANP-ZNF469*	T/C	−0.15	1.1 × 10^−11^	−0.07	0.0062	−0.11	4.2 × 10^−12^
rs4790881	17	2068932	*SMG6*	C/A	0.11	9.0 × 10^−12^	0.06	0.0100	0.10	1.3 × 10^−12^
rs34629349	17	4829560	*INCA1*	C/A	0.12	2.9 × 10^−13^	0.07	0.0063	0.11	2.2 × 10^−14^
rs9913911	17	10031183	*GAS7*	A/G	0.21	1.4 × 10^−42^	0.17	7.0 × 10^−12^	0.20	2.1 × 10^−52^
rs11659764	18	53335512	*TCF4-LINC01415*	T/A	0.26	4.0 × 10^−13^	0.20	0.0007	0.24	1.8 × 10^−15^
rs3918508	20	37912667	*LINC01734/LINC01370*	G/A	0.09	1.3 × 10^−9^	−0.02	0.42	0.06	3.1 × 10^−6^
rs2839082	21	47442334	*COL6A1-COL6A2*	C/T	0.09	3.5 × 10^−9^	0.04	0.14	0.07	7.8 × 10^−9^

SNP, single-nucleotide polymorphism; Chr, chromosome; GERA, Genetic Epidemiology Research in Adult Health and Aging; GWAS, genome-wide association studies; Pos, position; *β*, beta; *P*, *P* value

### Replication in an independent external cohort

We then tested the 40 lead single-nucleotide polymorphisms (SNPs) representing each of the 40 independent loci for replication in an independent external meta-analysis consisting of 37,930 individuals of European and Asian descent from 19 studies^11^. Of the 40 novel IOP-associated SNPs, 39 were associated with the same direction of effect in the replication sample, and 14 replicated at Bonferroni significance (*P* < 0.0013 = 0.05/40) (Table 2 **)**.

### Conditional and epistasis analyses

We next searched for additional genome-wide significant SNPs within a 2 Mb window (±1.0 Mb with respect to the lead SNP) including the 47 lead SNPs identified in the discovery GERA trans-ethnic GWAS analysis as covariates. We did not identify any additional genome-wide significant SNPs; however, we identified 12 SNPs with suggestive evidence of association with IOP that appeared to be independent signals (*P* < 0.0001 corresponding to an estimate of ~500 independent variants per locus for 2 Mb interval surrounding each of our original signals) (Supplementary Table 2). We then conducted an epistasis analysis of all pairs of lead SNPs in each race/ethnicity group, and we did not observe significant epistatic interactions between IOP SNPs after Bonferroni correction (*P* < 1.2 × 10^–5^  =  0.05/(((47*46)/2)*4)).

### Effect of the 47 IOP-associated loci on glaucoma

To investigate whether the 47 lead IOP-associated SNPs were also associated with primary open-angle glaucoma (POAG) susceptibility, we performed a case–control analysis in GERA including 2,338 POAG cases (normal and high-tension glaucoma) and 58,172 controls (Table 1). Forty-two of the 47 genome-wide significant IOP loci (89.4%) show a directionally consistent effect in the POAG GWAS. In particular, we found associations with glaucoma at a Bonferroni level of significance (*P* < 0.00106 = 0.05/47) for 6 SNPs. This included one SNP at a genome-wide level of significance for a previously identified locus at *TMCO1* (*P* = 2.8 × 10^−8^ for rs6668108) (Supplementary Table 3). The two lead SNPs for *TMCO1* (rs4656461 and rs7518099) previously reported to be associated with POAG at genome-wide significance^18, 19^, were strongly correlated with our lead SNP rs6668108 in European-Ancestry populations (*R* ^*2*^ = 0.99 and 1.0, respectively) (Supplementary Table 4). Other Bonferroni significant loci included *AFAP1* (*P* = 3.6 × 10^−6^ for rs28795989), *ABCA1* (*P* = 5.4 × 10^−6^ for rs2472493), and *GAS7* (*P* = 5.0 × 10^−7^ for rs9913911) (Supplementary Table 3). The two lead SNPs for *AFAP1* (rs4619890 and rs11732100) previously reported to be associated with POAG at genome-wide significance^18, 20^, were moderately correlated with our lead SNP rs28795989 in European-Ancestry populations (*R* ^*2*^ = 0.25 and 0.51, respectively) (Supplementary Table 4). Consistent with previous studies^18, 20^, we identified SNP rs2472493 as the lead SNP at the *ABCA1* locus. Our lead SNP rs9913911 in *GAS7* was relatively close (10.7 kb) to SNP rs9897123 previously reported^18^, and was in linkage disequilibrium (LD) (*R* ^2^ = 0.54, D’ = 0.95). We also found supportive evidence for the previously identified locus on *FOXC1* (*P* = 2.9 × 10^−4^) (Supplementary Table 3) with the same lead SNP rs2745572 reported by Bailey et al.^18^ (Supplementary Table 4). All above-mentioned alleles associated with higher IOP levels also raised glaucoma risk (Supplementary Table 3).

### Genetic association with related traits and eye diseases

In addition to glaucoma risk, several loci for which the associations with IOP reached genome-wide significance were already known to influence the variation of ocular traits, or eye diseases (Supplementary Table 4). These findings extend previous studies showing overlapping GWAS regions between POAG and related traits^8, 11, 18, 21, 22^. The current study identified a genome-wide significant signal with IOP at *EFEMP1* on chromosome 2. Common variants in *EFEMP1* (rs3791679 and rs1346786) have been shown to be associated with cup area, a specific optic disc measurement describing optic nerve morphology^11, 12^. Our lead SNP rs7426380 at *EFEMP1* was in LD with these two SNPs in European-Ancestry populations (*R* ^2^ = 0.33, *D*’ = 0.98 for rs3791679, and *R* ^2^ = 0.49, *D*’ = 0.98 for rs1346786) (Supplementary Table 4). Further, among our novel IOP loci, six have been previously associated with central corneal thickness, including *COL4A3*, *FAM46A-IBTK*, *ARID5B*, *FOXO1*, *SMAD3*, and *BANP-ZNF469* ^21, 23–26^. Except for SNP rs1538138 at *FAM46A-IBTK*, all the lead SNPs at those loci previously associated with central corneal thickness were in high LD with our lead SNPs in European-Ancestry populations (*R* ^2^ ranged from 0.97 to 0.99, and *D*’ ranged from 0.99 to 1.0) (Supplementary Table 4). We also identified a genome-wide significant signal with IOP on chromosome 9 at *GLIS3*, which is involved in the development of the eye^27^, and has been recently identified as a new susceptibility locus for primary angle-closure glaucoma^28^. Our lead SNP rs2224492 at *GLIS3* was correlated with the strongest SNP rs736893 reported by Khor et al.^29^ (*R* ^2^ = 0.71, *D*’ = 0.85), and the two SNPs were relatively close (20.5 kb apart). These findings suggest a shared genetic etiology between IOP and related traits; further investigations could help to better understand their shared mechanisms and biological pathways.

Our study also identified novel IOP loci that have been previously involved in autosomal dominant Mendelian ocular conditions. Indeed, deleterious mutations in *FOXC1* and *PITX2* can cause Axenfeld-Rieger syndrome, an anterior segment dysgenesis disorder characterized by anomalies in the anterior chamber angle, including defects in the drainage structures of the eye^30–32^. A major consequence of angle dysgenesis is an increase in IOP leading to the development of early-onset glaucoma, with *FOXC1* mutation carriers having a younger age at diagnosis in comparison with *PITX2* mutation carriers (6 vs. 18 years, respectively)^33^. Therefore, it is likely that common variants in *FOXC1* and *PITX2* mildly impact the drainage structures, and, in combination with defects of other genetic variants contribute to elevated IOP. Thus, it seems that there is a continuous spectrum of genetic predisposition to elevated IOP and related traits, from monogenic causative variants with high penetrance, to common polygenic variants with moderate to low penetrance.

### Gene expression in human ocular tissues

To prioritize genes for further investigation, we first identified the 95% credible set of variants in each of the 47 IOP loci (Supplementary Data 1—See Methods). We then examined expression levels of the genes in the 47 IOP loci that contained associated 95% credible set variants in human ocular tissues using two publicly available databases: the OTDB (Ocular Tissue Database)^34^, and EyeSAGE^35, 36^. According to the Ocular Tissue Database^34^, most of the identified genes were expressed in most ocular tissues, and some genes showed varied expression levels across ocular tissues (Supplementary Table 5). For instance, *ANTXR1* was highly expressed in the sclera, and *FMNL2* was highly expressed in the optic nerve and optic nerve head. *ANTXR1* and *FMNL2* were both novel loci identified in the present study which replicated in the external study. On average, the identified genes were most strongly expressed in the optic nerve head (mean ± SD: 90.2 ± 85.1), followed by the trabecular meshwork (mean ± SD: 82.5 ± 63.7) (Supplementary Table 5—Supplementary Fig. 2). The trabecular meshwork is located in the angle of the anterior chamber, and is responsible for draining the aqueous humor from the eye. Because of its role, it is a crucial determinant of IOP^37^.

### Gene prioritization and gene set enrichment analysis

To translate the 47 IOP genetic loci identified in the current study into biological insights, we used DEPICT^38^, which is not driven by phenotype-specific hypotheses. This integrative tool considers multiple lines of complementary evidence to systematically prioritize the most likely causal genes at associated loci, highlight enriched pathways, and identify tissues/cell types in which genes from associated loci are highly expressed. Gene prioritization analysis did not detect genes within the 47 identified loci to prioritize after false-discovery rate (FDR) correction; however, nominal evidence was found for seven genes, including *COL6A1*, *FOXO1*, *GLIS3*, *EFEMP1*, *CAV2*, *ANTXR1*, and *LPP* (Supplementary Table 6). Gene set enrichment analysis highlighted the “abnormal vascular endothelial cell morphology” pathway as the pathway in which the genes’ actions more likely affect IOP phenotype (*P* = 6.2 × 10^–9^; at FDR < 5%) (Supplementary Table 7). Tissue and cell type enrichment analysis detected a number of nominal enrichments (Supplementary Table 8).

### Replication of previous IOP GWAS results

We also investigated a total of 13 independent SNPs within 11 loci associated with IOP at a genome-wide significance level in previous studies^6–9, 11, 13, 22^ (Supplementary Table 9). Eight of the 13 replicated at a genome-wide level of significance in our GERA trans-ethnic meta-analysis (including *TMCO1* rs7555523, *FNDC3B* rs6445055, *CAV1* rs10258482 and rs423660, *ABCA1* rs2472493, *ARHGEF12* rs58073046, *GAS7* rs9913911 and rs11656696) and four at Bonferroni significance (*P* < 0.05/13 = 0.0038) (Supplementary Table 9). In contrast, *GLCCI1/ICA1* rs59072263 on chromosome 7, which was reported as genome-wide significant in a previous GWAS of IOP^6^, was not associated with IOP in the current study (*P* = 0.54).

### Heritability estimate and variance explained

Using GCTA^39^, we estimated array heritability in the non-Hispanic white sample to be 24.6% (SE = 1.0%). We then determined the proportion of variance explained by lead variants in each of the four race/ethnicity groups (Supplementary Table 10). We first estimated the variance explained using the 47 lead variants identified in the current study, which was highest in the non-Hispanic white group (3.66%), followed by East Asians (3.54%), Hispanic/Latinos (3.01%), and African-Americans (2.80%). For comparison, we conducted the same analysis for the previously reported lead SNPs, with the highest proportion of variance explained in East Asians (1.17%), non-Hispanic whites (1.09%), Hispanic/Latinos (0.84%), and African-Americans (0.25%). To determine how incorporating multiple measurements affected the proportion of variance explained, we estimated the variance explained by the 47 lead variants using a single, randomly chosen IOP measurement for each individual in the sample. The proportion of variance explained decreased in each group to 2.88% in non-Hispanic whites, 2.58% in East Asians, 2.60% in Hispanic/Latinos, and 2.65% in African-Americans.

## Discussion

In the large, ethnically diverse GERA cohort, we discovered 40 novel genome-wide significant IOP loci, of which 14 replicated at Bonferroni level of significance in an independent external meta-analysis^11^. We further examined their effect on the risk of glaucoma within our discovery sample, and summarized genetic associations at these IOP loci with other vision disorders and related traits previously reported in the literature. We also confirmed the association of variants with IOP in 10 loci previously reported, including *TMCO1*, *FNDC3B*, *CAV1*, *ABCA1*, *FAM125B/LMX1B*, *ABO*, *ARHGEF12*, *ADAMTS8*, *GAS7,* and on chromosome 11p11.2 encompassing several genes^7–9, 11, 13, 22^.

Our findings extend previous studies showing the important role for specific components of the drainage structure in controlling IOP^4, 37^. The meshwork is primarily composed of three layers, with the outermost region of the trabecular meshwork lined by the endothelial cells, forming the inner wall of Schlemm’s canal (a modified capillary blood vessel that forms intra- and intercellular pores). Aqueous humor passes through these different tissue layers and flows into the Schlemm’s canal. Consistent with a role of the drainage tissue in IOP regulation, some of the genes identified in the current study were, on average, more expressed in the trabecular meshwork than other ocular tissues (e.g., *TNS1*, *LPP*, *GLIS3*, and *FOXO1*). To date, the cellular signaling pathways that maintain the outflow of aqueous humor and IOP homeostasis are not well understood^40, 41^. Resistance to aqueous humor flow into the Schlemm’s canal maintains the physiological IOP of the eye. Although the identity of cell types and tissue layers supporting conventional outflow is well established, the specific contribution of the individual layers toward generating resistance to aqueous flow remains unclear. Based on current views, resistance to aqueous humor outflow is imparted at the region just proximal to the Schlemm’s canal^42–44^. In the disease state, increased resistance to aqueous humor outflow is thought to contribute to elevated IOP. Our in silico results provide compelling evidence that support “abnormal vascular endothelial cell morphology” as the pathway in which the genes’ actions at the identified loci more likely affect IOP. Together, these findings suggest a potential role for Schlemm’s canal endothelial cells in imparting resistance to aqueous humor outflow and thereby influencing IOP regulation.

The current study also highlights potential strong candidate genes for further investigation among the novel IOP loci identified, which replicated in the external sample, with substantial biological evidence from the literature and our in silico results. Some of these genes belong to cellular signaling pathways critical for IOP regulation, and involving components of the extracellular matrix (ECM), and endothelial cell function^4, 40, 41^. *ANTXR1* encodes a type I transmembrane protein known as anthrax toxin receptor 1, which is involved in the vascular endothelial growth factor signaling and ECM synthesis to control mechanisms of connective tissue development and homeostasis^45, 46^. Similarly, *TCF4*, that encodes transcription factor 4, is a major risk factor for Fuchs endothelial corneal dystrophy, a corneal disease leading to visual impairment and blindness^47–50^, and owing, notably, to an excessive accumulation of ECM^51^. Thus, mutations in *ANTXR1* and *TCF4* could contribute to increased resistance, through an excessive synthesis or accumulation of ECM in the ocular drainage tissue, thereby impeding flow of aqueous humor and resulting in elevated IOP.

Our study also led to the identification of genes belonging to cellular signaling pathways linked to cellular contraction and adhesion, and cell–cell, cell–ECM contact or adhesion^4, 40, 41^. Cellular contraction and relaxation is thought to modulate permeability of trabecular meshwork cells and aqueous humor outflow^52^. *FMNL2* encodes a formin-related protein, named formin like 2, and formin-related proteins have been implicated in actin nucleation and elongation as well as in lamellipodia protrusion^53^. Because of its action on the cytoskeleton, FMNL2 could have a crucial role in the contractile mechanism in the conventional outflow pathways and adjoining layers, affecting the outflow resistance and IOP. Thus, we speculate that mutations in this gene could modify the contractile properties of the tissue layers that make up the drainage structure of the eye, resulting in IOP modulation. Follow-up experiments of this potential candidate gene using in vivo animal models would aid in our understanding of its role in the regulation of IOP and glaucoma development.

Our study was limited by its restriction to common variants (minor allele frequency ≥ 1%), which did not allow us to identify lower frequency variants that contribute to variation in IOP. Indeed, a previous study has conducted an exome-wide association analysis and identified less common variants associated with IOP, in particular in the CAV1/CAV2 region^7^. In addition, our study was limited in its replication of prior GWAS findings by the smaller size of the replication cohort available, somewhat reducing confidence in the novel associations found in our larger discovery cohort. This concern is mitigated by findings from our case–control analysis of glaucoma, which show that 15 of the novel loci associated with IOP are also associated with glaucoma at Bonferroni or nominal level of significance, including three loci (*AFAP1*, *FOXC1*, and *PKHD1*) previously found in association with glaucoma. In addition, loci associated with IOP were associated with a number of other eye conditions and traits consistent with a plausible role in affecting IOP. These limitations are mitigated by some significant strengths, beginning with the use of the very large, generally representative GERA cohort as the basis for the study. The GERA cohort comprises an unselected sample of adults (i.e., not selected for vision disorders or other health conditions) drawn from members of a health care delivery system whose members are representative of the general population in northern California. Vision care is included for all members of the Kaiser Permanente Health Plan and IOP measurement is a standard component of vision examinations, making it unlikely that the findings of the study are owing to selection bias, such as the inclusion of IOP measurements from a less “healthy” sample of eyes or individuals seeking care for glaucoma or other eye conditions. We also focused on measurements taken on subjects prior to any IOP lowering medication treatments and selected the median value of the mean IOP of both eyes. Further, our study is based on a very large, demographically representative and ethnically diverse cohort, with multiple IOP measurements available for each participant, providing greater precision of measurement, and consequently statistical power, than if only a single measurement was taken. Further, the genotyping arrays had more extensive genomic coverage than previously used arrays in published studies, and the 1000 genomes imputation reference panel was larger than those used in many previous studies, further enhancing power to discover IOP genetic loci.

In summary, the current study demonstrates the utility of using multiple phenotype measurements from electronic health records in combination with a large and diverse sample for identifying novel genetic loci underlying a complex trait such as IOP. Our report of 40 novel loci associated with IOP substantially increased the proportion of variance in IOP explained by specific genetic factors. This study is an important step toward understanding the mechanisms underlying IOP regulation and, consequently, glaucoma risk and progression.

## Methods

### Study population

We report a genome-wide association study (GWAS) of IOP in 69,756 individuals from the GERA cohort. The GERA cohort comprises 110,266 adult men and women who are consented participants in the Research Program on Genes, Environment, and Health, drawn from members of the Kaiser Permanente Medical Care Plan, Northern California Region (KPNC); the GERA cohort has been described in detail elsewhere^54, 55^. The current study population consisted of 69,756 adults, 18 years and older, who were of non-Hispanic white, Hispanic/Latino, Asian, or African American race/ethnicity, and who had at least one recorded IOP measurement on both eyes during the same visit between January 2006 and January 2017 (Table 1). All study procedures were approved by the Institutional Review Board of the Kaiser Foundation Research Institute.

### Phenotype definition

IOP measurements, as entered by clinicians at each vision encounter, were captured in the electronic health records as smart variables. The main standard equipment for measuring IOP in KPNC ophthalmology practices is a Goldmann applanation tonometer (Haag-Streit, Bern, Switzerland), followed by a non-contact tonometer (Nidek TonoRef II), a Tono-Pen XL, an iCare rebound tonometer (Tiolat, Helsinki, Finland) and other equipment, including pneumotonometers (Supplementary Table 11). For this analysis, we removed non-numeric entries, extreme IOP values (≤5 and >60 mmHg), measurements taken on a single eye, and 116,980 IOP measurements from 3,632 participants that were taken after prescription of IOP lowering medications to exclude values influenced by treatment. When there was more than one IOP measurement on a single day, if different methods were used, we chose the highest quality measurement based on the method used (Goldmann applanation tonometer >iCare rebound tonometer >non-contact tonometer >Tono-Pen XL >other/pneumotonometer)^16^. If the same method was used for multiple measurements on the same day, we took the mean of all measurements. Finally, individual’s mean IOP from both eyes for each visit was assessed, and the individual’s median of the mean across all the visits was used for analysis (Fig. 1). In total, 356,987 IOP measurements were included in this study.

### Glaucoma cases and controls

Among the 69,756 participants included in the current IOP study, 2,338 have been diagnosed with “glaucoma” clinically (Table 1). We defined “glaucoma” as having at least: two diagnoses of POAG, or two diagnoses of normal tension glaucoma, or one diagnosis of POAG and one diagnosis of normal tension glaucoma. In all cases, at least one of the diagnoses was made by a Kaiser Permanente ophthalmologist. For the control group, participants who had one or more diagnosis of any type of glaucoma (e.g., pseudoexfoliation, pigmentary, or PACG) were excluded. The final control sample included 58,172 participants.

### Genotyping and imputation

DNA samples from GERA individuals were extracted from Oragene kits (DNA Genotek Inc., Ottawa, ON, Canada) at KPNC and genotyped at the Genomics Core Facility of the University of California, San Francisco (UCSF). DNA samples were genotyped at over 665,000 SNPs on four race/ethnicity-specific Affymetrix Axiom arrays (Affymetrix, Santa Clara, CA, USA) optimized for individuals of European, Latino, East Asian, and African-American ancestry^56, 57^. Genotype QC (quality control) procedures for the GERA samples were performed on an array-wise basis^55^. SNPs with initial genotyping call rate ≥97%, allele frequency difference (≤0.15) between males and females for autosomal markers, and genotype concordance rate (>0.75) across duplicate samples were included. About 94% of samples and more than 98% of genetic markers assayed passed QC procedures. In addition to those QC criteria, SNPs with genotype call rates <90% were removed, as well as SNPs with a minor allele frequency <1%.

Imputation was also conducted on an array-wise basis. Following the pre-phasing of genotypes with Shape-IT v2.r72719^58^, variants were imputed from the cosmopolitan 1000 Genomes Project reference panel (phase I integrated release; http://1000genomes.org) using IMPUTE2 v2.3.0^59–61^. As a QC metric, we used the info *r* ^*2*^ from IMPUTE2, which is an estimate of the correlation of the imputed genotype to the true genotype^62^. We excluded variants with an imputation *r* ^2^ < 0.3, and restricted to SNPs that had a minor allele count ≥20.

### GWAS analysis and covariate adjustment

We first analyzed each of the four race/ethnicity groups (non-Hispanic whites, Hispanic/Latinos, East Asians, and African-Americans) separately. We performed a linear regression of each individual’s median of the mean IOP with the following covariates: age at the median measurement, sex, and ancestry principal components (PCs) (Supplementary Table 12). We then performed a linear regression of the residuals on each SNP using PLINK^63^ v1.9 (www.cog-genomics.org/plink/1.9/) to assess genetic associations. Data from each SNP were modeled using additive dosages to account for the uncertainty of imputation^64^.

Eigenstrat^65^ v4.2 was used to calculate the PCs on each of the four race/ethnicity groups^54^. The top 10 ancestry PCs were included as covariates for the non-Hispanic whites, while the top six ancestry PCs were included for the three other race/ethnicity groups. The percentage of Ashkenazi ancestry was also used as a covariate for the non-Hispanic whites to adjust for genetic ancestry, as described previously^54^.

Second, we undertook a GERA meta-analysis of IOP to combine the results of the four race/ethnicity groups using the R^66^ (https://www.R-project.org) package “meta”. We calculated fixed effects summary estimates under an additive model, and we assessed heterogeneity index, *I* ^2^ (0–100%) among groups as well as Cochran’s *Q* heterogeneity statistic. At each locus, the lead SNP was defined as the most significant SNP within a 2 Mb window. New loci were defined as those that were located more than 1 Mb apart from any previously described locus.

Finally, to identify additional independent SNPs at each locus, we conducted association analyses by including all the 47 lead SNPs identified in the GERA trans-ethnic meta-analysis as covariates in the regression model. We assessed whether any additional SNPs within a 2 Mb window ( ± 1.0 Mb with respect to the original lead SNP) reached genome-wide significance. We report associations that replicate at a Bonferroni-corrected significance threshold of 0.05/500 = 0.0001 (corresponding to an estimate of ~500 independent variants per locus for 2 Mb interval surrounding each of our original signals), as previously used^67^. An epistasis analysis of all pairs of lead SNPs was also conducted in the four GERA race/ethnicity groups (non-Hispanic whites, Hispanic/Latinos, East Asians and African/Americans). For this analysis, we applied a Bonferroni-corrected significance threshold of 0.05/4,324 = 1.2 × 10^–5^ (accounting for the number of SNP-pairs tested (47*46)/2, and for the four race/ethnicity groups).

### Glaucoma case–control analysis

We evaluated the associations of the 47 lead IOP-associated SNPs with glaucoma susceptibility by logistic regression under an additive model, and adjusting for age, sex, and ancestry PCs.

### Replication of novel SNPs in an independent external cohort

To test the 40 novel GERA genome-wide significant SNPs for replication, we evaluated associations in an independent external study. GWAS summary statistics from the study of Springelkamp et al.^11^, consisting of 37,930 individuals of European and Asian descent from 19 studies, were publicly accessible. We also combined the results for the 40 novel identified SNPs using a meta-analysis of GERA and the study of Springelkamp et al.^11^. We report fixed effects results, and associations that replicate at a strict Bonferroni threshold (*P* < 0.00125, to account for a total of 40 SNPs tested).

### Replication analysis of previously reported SNPs in GERA

To determine how many of the 11 previously reported IOP loci from genetic studies replicated in the GERA cohort, we tested 13 statistically independent lead SNPs previously reported to be associated at a genome-wide level of significance^6–9, 11, 13, 22^. We used a nominal significance level of 0.05, and a more stringent multiple testing correction accounting for the number of SNPs tested (Bonferroni-corrected alpha level of 0.0038 ( = 0.05/13)).

### GWAS heritability estimates and variance explained

SNP-based heritability estimates were obtained for IOP using the GCTA software^39^, which computes the phenotypic variance explained by all analyzed SNPs in the genome by restricted maximum likelihood achieved using expectation maximization. We restricted the analysis to autosomal SNPs, and a genetic relationship matrix cutoff of 0.025 was applied. For statistical power purposes, we conducted the analysis in the largest group of individuals from GERA, which is the non-Hispanic white.

We also estimated the variance explained by (1) the 47 lead SNPs identified in the current study; and (2) the 13 SNPs previously identified, using a linear regression on the IOP residuals, and including either the 47 lead SNPs or the 13 previously reported SNPs as covariates in the model. To assess the impact of multiple IOP measurements on the proportion of variance explained, we also estimated the variance explained by the 47 lead SNPs using a single, randomly selected IOP measurement for each individual.

### In silico analyses

To produce the most thorough list of candidate genes within the 47 identified loci, we used a Bayesian approach (CAVIARBF)^68^, publicly available at https://bitbucket.org/Wenan/caviarbf. Briefly, for each of the 47 signals, we computed each variant’s ability to explain the observed signal within a 2 Mb window (±1.0 Mb with respect to the original lead SNP) and derived, the smallest set of variants that included the causal variant with 95% probability (95% credible set). Previous studies^69, 70^ have used similar approaches to prioritize variants near index SNPs for follow-up. These 47 credible sets included a total of 12,614 variants in 59 annotated genes (Supplementary Data 1).

Expression of the genes that contained associated 95% credible set variants was assessed in human ocular tissues using two publicly available databases: the OTDB^34^, and EyeSAGE^35, 36^ publicly available at https://genome.uiowa.edu/otdb/, and http://neibank.nei.nih.gov/EyeSAGE/index.shtml, respectively. The OTDB contains gene expression data for 10 eye tissues from 20 normal human donors, and the gene expression is reported as Affymetrix Probe Logarithmic Intensity Error normalized value, as previously described^34^.

We then prioritized potentially causal genes for the 47 associations identified in the GERA GWAS using DEPICT^38^, a previously described bioinformatics tool that is not driven by phenotype-specific hypotheses. All the 47 lead SNPs that achieved *P* < 5 × 10^–8^ in the GERA GWAS served as input, and information on prioritized genes was extracted. Genes that reached a nominal significance level of 0.05 in DEPICT were subsequently prioritized. Finally, enriched gene set/pathways and tissues/cell types were highlighted using DEPICT^38^ and the same 47 lead SNPs input.

### Data availability

Genotype data of GERA participants are available from the dbGaP (database of Genotypes and Phenotypes) under accession phs000674.v2.p2. This includes individuals who consented to having their data shared with dbGaP. The complete GERA data are available upon application to the KP Research Bank (https://researchbank.kaiserpermanente.org/). The summary statistics generated in this study are available from the corresponding authors upon reasonable request. The GWAS summary statistics for the replication study^11^ are available from (https://www.dropbox.com/sh/3j2h9qdbzjwvaj1/AABFD1eyNetiF63I5bQooYura?dl¼0).

## Electronic supplementary material

Supplementary Information

Description of Additional Supplementary Files

Supplementary Data 1

Peer Review File
