# Supplementary material for: A large multi-ethnic genome-wide association study identifies novel genetic loci for intraocular pressure
Source: Nat Commun. 2017 Dec 13;8:2108. doi: 10.1038/s41467-017-01913-6 (PMC5727399; doi:10.1038/s41467-017-01913-6)
Supplement: Supplementary file 1 — Supplementary Information [file 41467_2017_1913_MOESM1_ESM.pdf]

Supplementary Figures and Tables

Supplementary Figure 1. QQ plots and genomic inflation factors (λ) observed in **a.** the discovery GERA trans-ethnic meta-analysis of IOP, and each race/ethnicity group: **b.** non-Hispanic white, **c.** Hispanic/Latino, **d.** East Asian, and **e.** African-American

a.

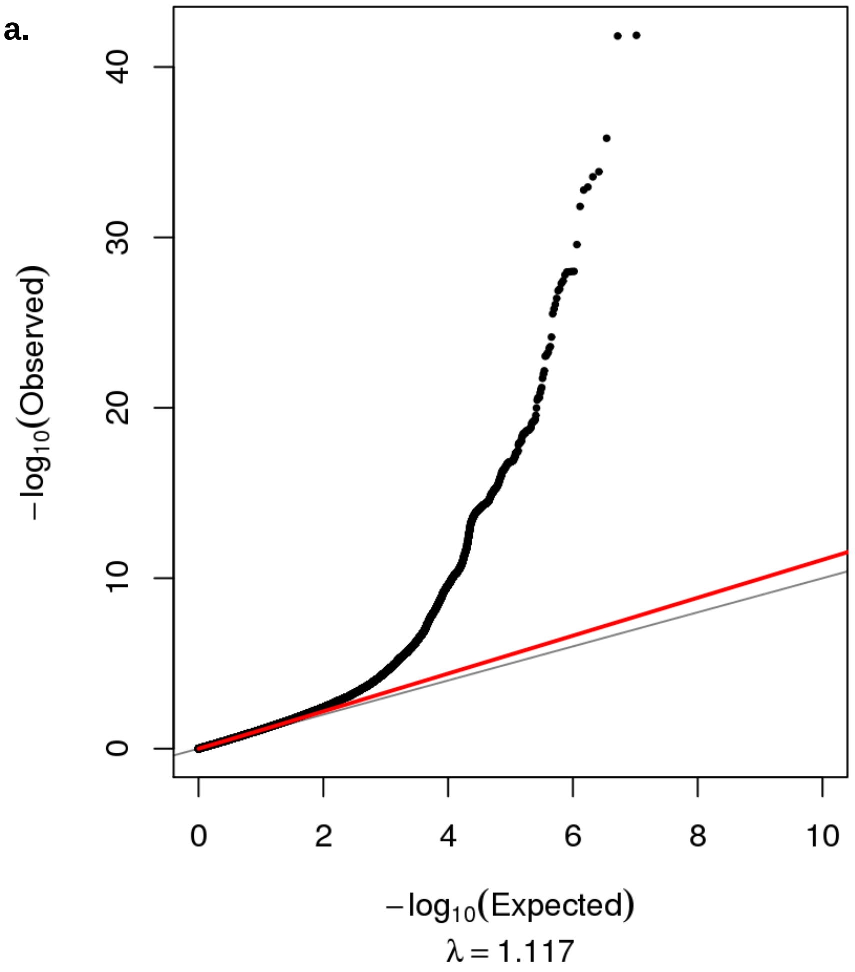

b.

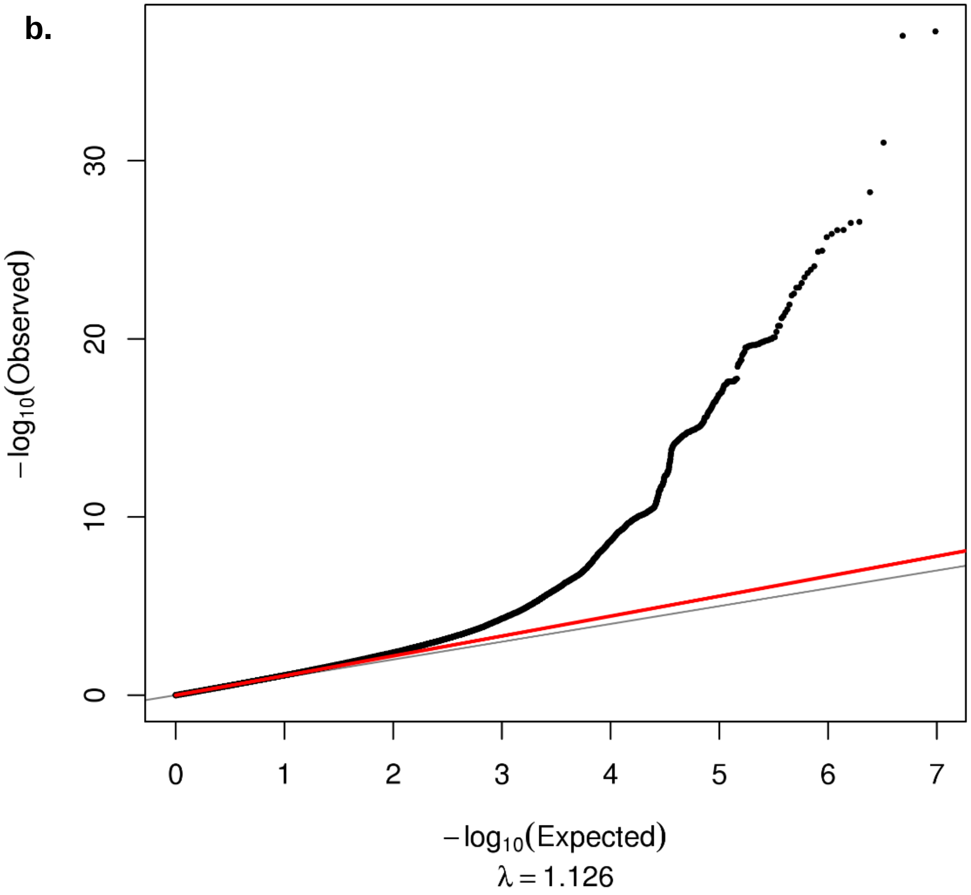

c.

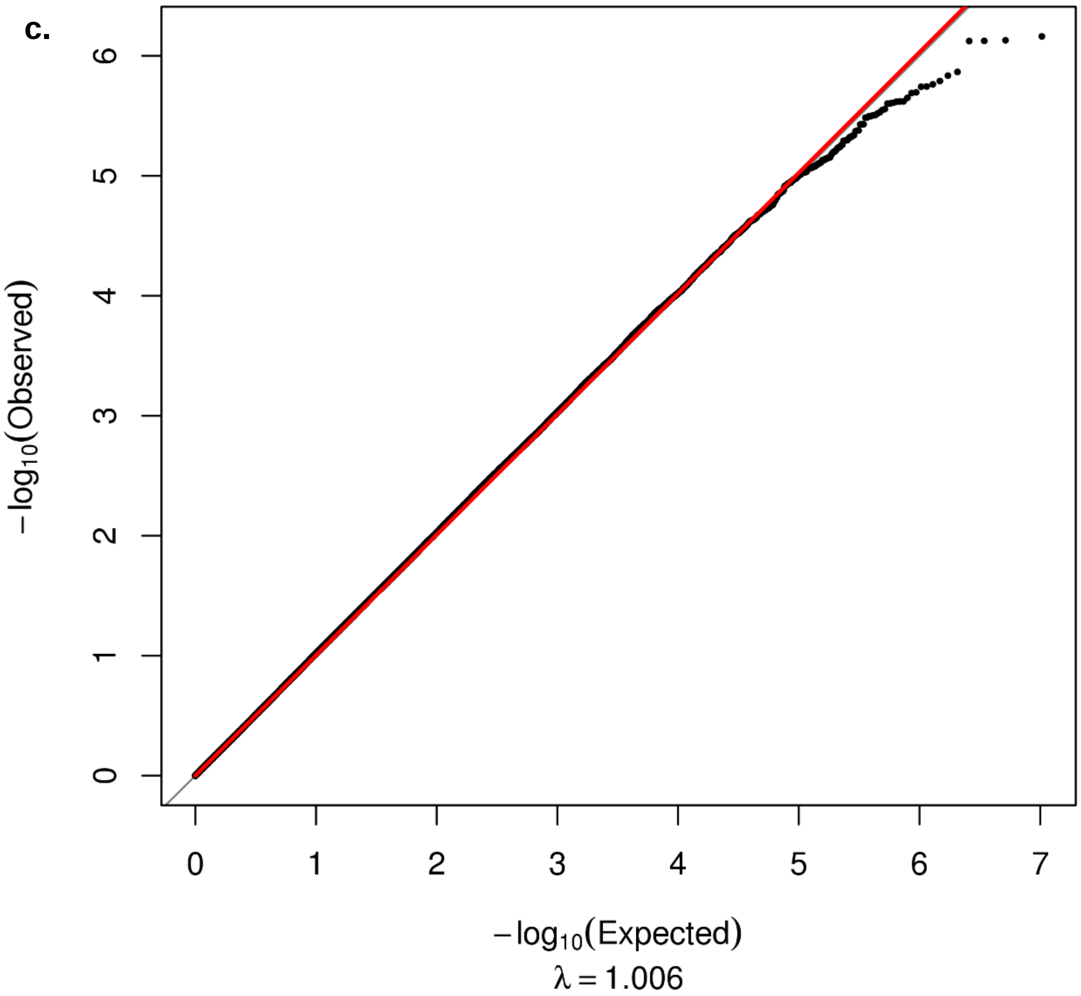

d.

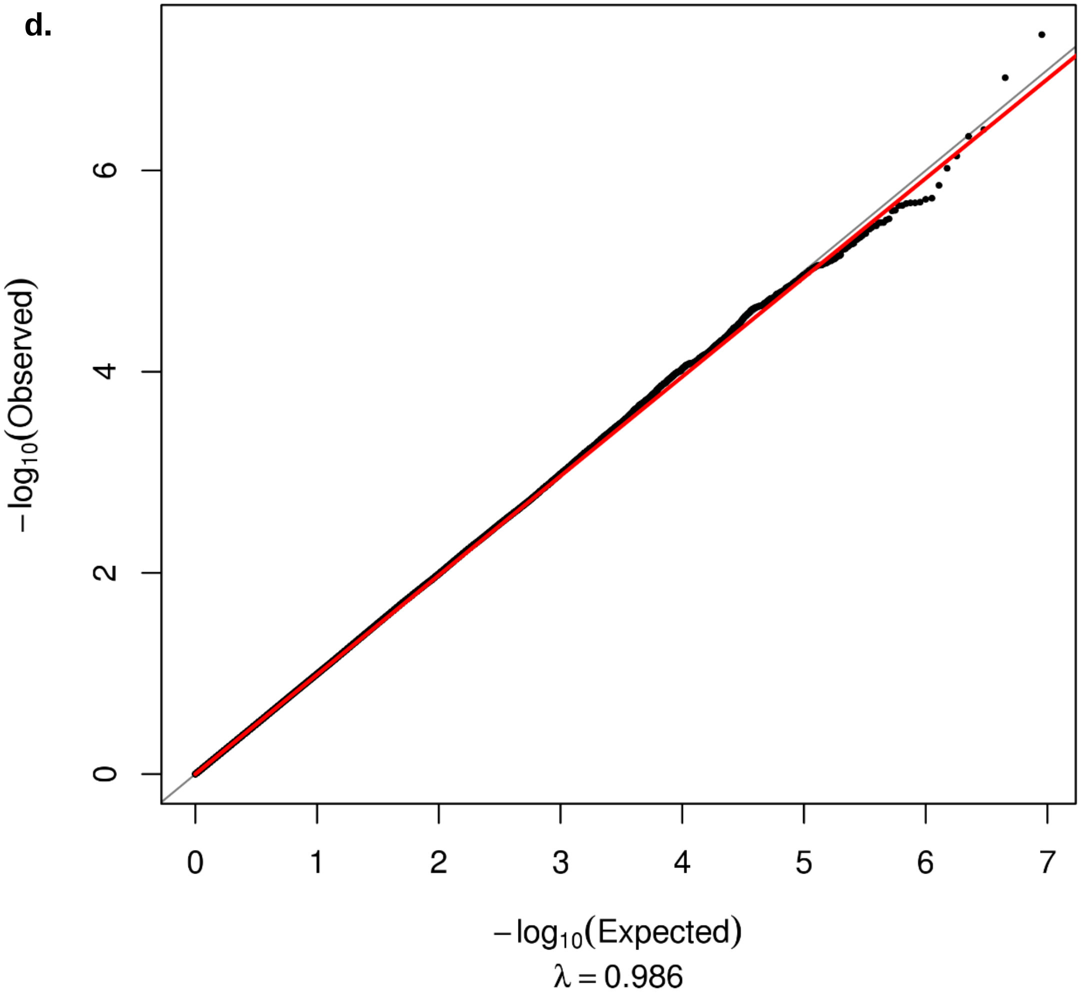

e.

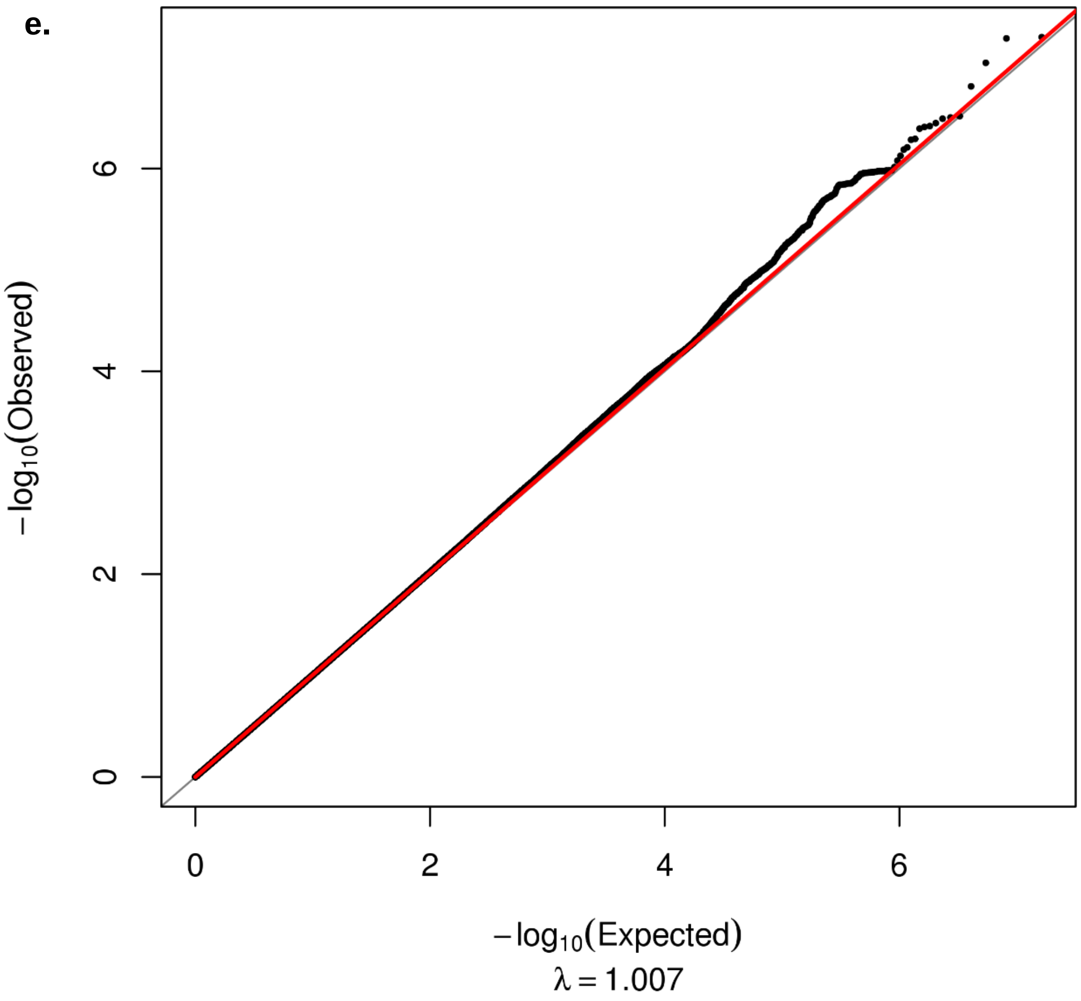

Supplementary Figure 2. Expression of genes in IOP loci that contained associated 95% credible set variants in adult human eye tissues according to the Ocular Tissue Database[1] (OTDB)

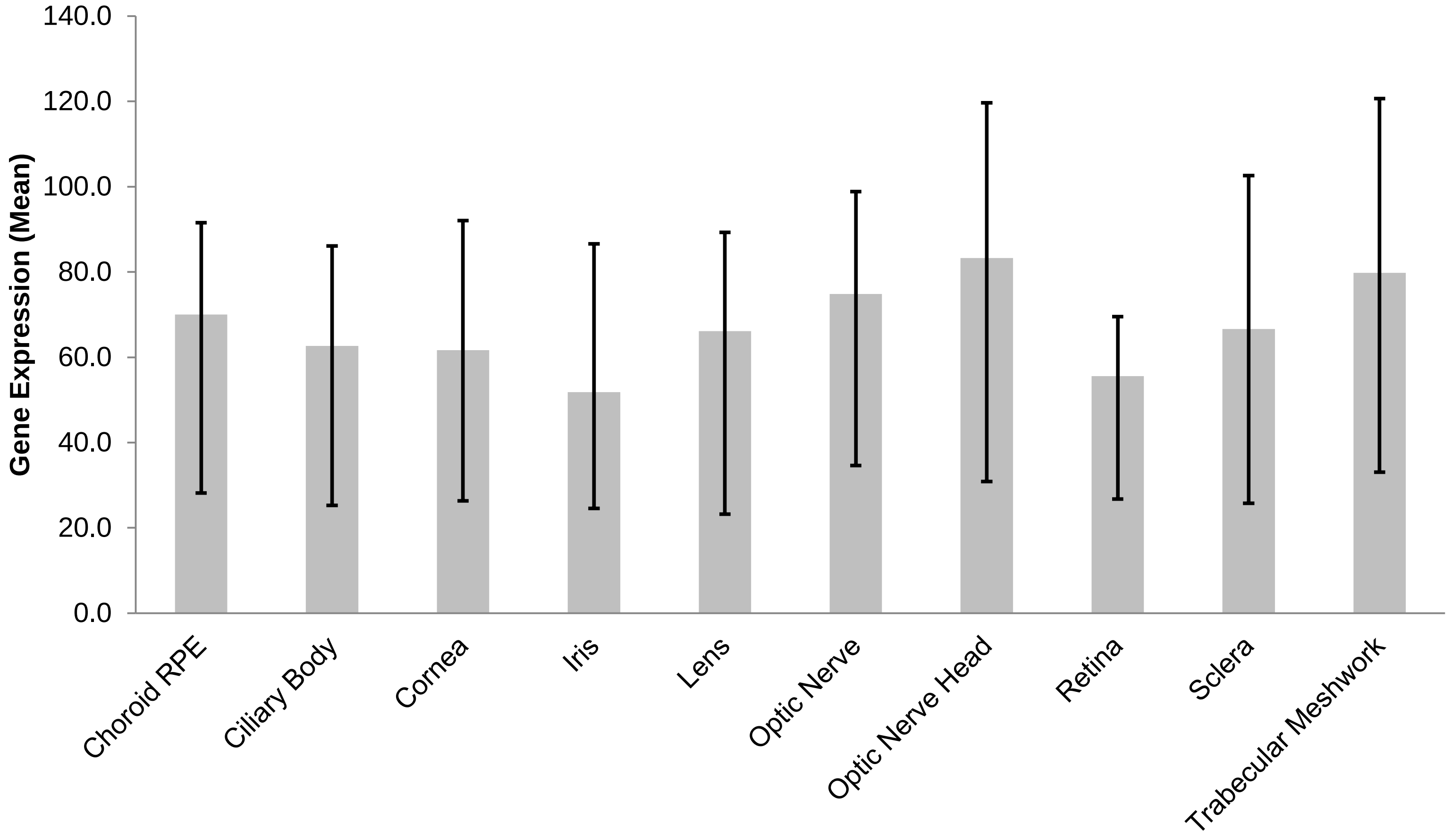

The gene expression is indicated as Affymetrix Probe Logarithmic Intensity Error (PLIER) number Vertical; error bars correspond to the interquartile range (IQR);

Supplementary Table 1. Lead IOP SNPs ($P < 5 \times 10^{-8}$) by race/ethnicity group in the GERA discovery cohort

			NHW			H/L			EAS			AFR			META	
**SNP**	**Chr**	**Alleles**	**Frq**	**β**	***P***	**Frq**	**β**	***P***	**Frq**	**β**	***P***	**Frq**	**β**	***P***	***P*het**	**I^2**
rs1866758	1	T/C	0.62	0.10	**2.9×10^{-8}**	0.68	0.10	0.068	0.82	0.09	0.15	0.67	0.08	0.39	1.00	0.0
rs6668108	1	A/G	0.12	0.24	**1.9×10^{-21}**	0.13	0.13	0.096	0.02	-0.10	0.63	0.08	0.01	0.96	0.09	53.5
rs596169	1	A/G	0.07	-0.18	6.0×10^{-8}	0.07	-0.19	0.053	0.01	NA	NA	0.42	-0.18	0.062	0.99	0.0
rs115179432	2	A/G	0.93	-0.21	**1.2×10^{-10}**	0.94	-0.07	0.53	1.00	NA	NA	0.96	0.01	0.98	0.35	5.2
rs7426380	2	A/G	0.54	-0.10	**3.4×10^{-10}**	0.59	-0.13	0.013	0.13	-0.09	0.25	0.33	-0.13	0.19	0.97	0.0
rs6732795	2	A/C	0.61	-0.10	**3.5×10^{-9}**	0.55	-0.12	0.016	0.18	-0.07	0.28	0.31	0.02	0.83	0.62	0.0
rs55692468	2	T/G	0.39	-0.09	2.7×10^{-7}	0.37	-0.11	0.033	0.84	-0.06	0.40	0.72	-0.15	0.13	0.84	0.0
rs1035673	2	T/C	0.39	-0.09	1.9×10^{-7}	0.38	-0.16	0.002	0.50	-0.10	0.050	0.25	-0.06	0.57	0.62	0.0
rs143937055	2	TTTG/T	0.66	0.09	1.0×10^{-7}	0.63	0.10	0.060	0.61	0.14	0.010	0.77	-0.02	0.88	0.65	0.0
rs7599762	2	G/C	0.16	-0.14	**5.8×10^{-9}**	0.11	-0.01	0.89	0.04	-0.24	0.083	0.05	-0.08	0.73	0.46	0.0
rs11710845	3	C/T	0.75	0.11	**3.7×10^{-8}**	0.83	0.08	0.23	0.92	0.05	0.62	0.89	0.24	0.12	0.73	0.0
rs7635832	3	T/G	0.82	0.24	**5.8×10^{-29}**	0.77	0.24	0.0001	0.71	0.23	4.5×10^{-5}	0.74	0.19	0.079	0.96	0.0
rs9853115	3	T/A	0.49	0.11	**9.8×10^{-11}**	0.47	0.16	0.002	0.25	0.11	0.077	0.51	0.01	0.94	0.51	0.0
rs13076750	3	A/G	0.74	0.12	**1.5×10^{-10}**	0.68	0.03	0.58	0.57	0.19	0.0003	0.45	0.13	0.18	0.24	28.4
rs28795989	4	A/G	0.43	0.15	**3.6×10^{-19}**	0.59	0.07	0.19	0.84	0.03	0.62	0.79	-0.03	0.80	0.07	57.1
rs17527016	4	C/T	0.76	0.12	**2.6×10^{-10}**	0.85	-0.07	0.35	0.93	0.04	0.71	0.93	0.08	0.65	0.07	58.2
rs4865762	5	C/A	0.52	0.11	**9.8×10^{-12}**	0.53	0.10	0.055	0.79	0.11	0.067	0.59	0.24	0.013	0.62	0.0
rs73220188	5	T/C	0.88	0.14	**3.1×10^{-8}**	0.92	0.13	0.16	0.93	0.27	0.008	0.91	0.27	0.11	0.55	0.0
rs2745572	6	A/G	0.65	0.09	9.6×10^{-8}	0.62	0.17	0.002	0.51	0.17	0.002	0.80	0.19	0.11	0.33	11.7
rs1396046	6	G/A	0.36	-0.09	4.1×10^{-7}	0.51	-0.10	0.056	0.47	-0.15	0.004	0.75	-0.16	0.12	0.66	0.0
rs2875087	6	C/T	0.44	0.12	**9.6×10^{-13}**	0.46	0.13	0.012	0.46	0.14	0.005	0.26	0.30	0.005	0.41	0.0
rs141917145	6	AAAGA AACAA G/A	0.23	0.09	1.6×10^{-5}	0.29	0.10	0.090	0.43	0.19	0.0002	0.25	0.16	0.13	0.25	27.0
rs1509922	7	G/A	0.57	0.11	**2.4×10^{-10}**	0.43	0.05	0.35	0.16	0.10	0.15	0.63	0.16	0.10	0.67	0.0
rs6968419	7	A/G	0.60	0.14	**9.9×10^{-16}**	0.58	0.12	0.021	0.21	0.15	0.018	0.51	0.01	0.89	0.61	0.0
rs10105844	8	G/A	0.23	-0.12	**9.3×10^{-9}**	0.22	0.03	0.63	0.49	-0.09	0.095	0.16	-0.11	0.40	0.16	42.1
rs2224492	9	A/G	0.66	-0.13	**1.5×10^{-13}**	0.67	-0.14	0.015	0.68	-0.11	0.047	0.89	-0.21	0.16	0.94	0.0
rs1831902	9	C/T	0.21	-0.12	**4.3×10^{-9}**	0.17	-0.15	0.028	0.21	-0.10	0.12	0.32	0.03	0.75	0.48	0.0
rs2472493	9	G/A	0.42	0.18	**1.3×10^{-26}**	0.37	0.10	0.053	0.56	0.12	0.023	0.35	0.17	0.072	0.37	4.9
rs11014632	10	A/G	0.51	0.10	**2.8×10^{-9}**	0.47	0.02	0.66	0.26	0.02	0.6947	0.22	-0.04	0.71	0.20	35.5
rs5785510	10	CA/C	0.58	0.11	**1.2×10^{-10}**	0.66	0.04	0.46	0.64	0.13	0.019	0.80	0.28	0.018	0.28	21.3
rs66479974	10	CAG/C	0.39	0.09	6.6×10^{-7}	0.50	0.09	0.088	0.83	0.07	0.31	0.53	0.17	0.075	0.85	0.0
rs79390637	11	C/A	0.30	0.12	**1.0×10^{-10}**	0.34	0.24	2.0×10^{-5}	0.41	0.17	0.0010	0.56	0.19	0.052	0.21	34.0

rs199800298	11	AG/A	0.60	-0.12	**8.1×10^{-12}**	0.62	-0.07	0.19	0.67	-0.20	0.0003	0.42	-0.33	0.0005	0.05	61.9
rs7977237	12	T/A	0.33	0.09	4.3×10^{-7}	0.27	0.08	0.16	0.02	0.25	0.12	0.11	0.20	0.18	0.68	0.0
rs74481774	12	A/G	0.98	0.28	1.6×10^{-5}	0.98	0.32	0.087	0.99	NA	NA	0.87	0.47	0.001	0.47	0.0
rs9552680	13	C/T	0.69	-0.09	2.8×10^{-7}	0.73	-0.09	0.13	0.65	-0.08	0.12	0.64	-0.06	0.54	0.99	0.0
rs11616662	13	G/A	0.89	0.19	**5.9×10^{-13}**	0.89	0.36	2.2×10^{-5}	0.99	NA	NA	0.86	0.24	0.074	0.18	40.9
rs12912045	15	C/T	0.78	-0.10	2.2×10^{-7}	0.73	-0.22	0.0001	0.59	-0.20	0.0003	0.75	0.20	0.077	0.00	78.3
rs72755233	15	G/A	0.90	-0.25	**1.6×10^{-19}**	0.95	-0.22	0.051	0.99	NA	NA	0.97	0.30	0.29	0.15	46.9
rs75828804	16	G/A	0.92	-0.17	**6.7×10^{-9}**	0.86	-0.22	0.003	0.98	-0.22	0.29	0.94	-0.28	0.14	0.89	0.0
rs12926024	16	T/C	0.34	-0.15	**6.6×10^{-10}**	0.27	-0.14	0.017	0.07	-0.16	0.19	0.34	-0.09	0.34	0.96	0.0
rs4790881	17	C/A	0.29	0.10	**1.0×10^{-8}**	0.24	0.07	0.26	0.57	0.19	0.0001	0.15	0.15	0.26	0.34	10.9
rs34629349	17	C/A	0.34	0.13	**2.3×10^{-13}**	0.39	0.09	0.08	0.79	0.03	0.64	0.46	0.02	0.84	0.29	20.1
rs9913911	17	A/G	0.62	0.22	**5.5×10^{-38}**	0.67	0.10	0.055	0.52	0.23	6.0×10^{-6}	0.79	0.17	0.14	0.20	35.4
rs11659764	18	T/A	0.95	0.27	**2.8×10^{-13}**	0.97	0.05	0.75	1.00	NA	NA	0.99	0.62	0.15	0.25	27.6
rs3918508	20	G/A	0.48	0.09	**2.1×10^{-8}**	0.35	0.11	0.034	0.26	0.04	0.47	0.42	0.10	0.27	0.82	0.0
rs2839082	21	C/T	0.55	0.09	**8.8×10^{-8}**	0.48	0.08	0.13	0.33	0.08	0.15	0.78	0.17	0.12	0.89	0.0

NHW: non-Hispanic whites; H/L: Hispanic/Latinos; EAS: East Asians; AA: African-Americans; β, beta; Frq, frequency of effect allele; *P*het, heterogeneity *P*-values from Cochrane's Q statistic; I^2, heterogeneity index (0–100%); P-values in bold achieved genome-wide level of significance ($P < 5 \times 10^{-8}$);

Supplementary Table 2. Conditional analysis results from the GERA trans-ethnic meta-analysis

**SNP**	**Chr**	**Pos**	**Locus**	**Alleles**	**β**	***P***
rs2377923	1	218662465	*MIR548F3*	C/T	-0.16	3.1×10^{-5}
rs71320328	3	185715428	*DGKG-LINC02052*	A/T	0.10	2.8×10^{-5}
rs73190802	3	188025762	*LPP*	G/A	0.22	8.1×10^{-6}
rs149832919	5	52739179	*MOCS2/FST*	TTC/T	0.18	4.3×10^{-5}
rs115312654	6	50954097	*PKHD1*	A/T	0.61	6.5×10^{-5}
rs5745745	7	81336535	*HGF*	C/A	0.12	6.7×10^{-5}
rs111619203	12	44137342	*PUS7L*	G/A	-0.30	6.5×10^{-6}
rs1782796	13	40983815	*LINC00598*	C/T	-0.32	7.6×10^{-6}
rs75040933	16	76836381	*ADAMTS18-NUDT7*	C/T	-0.25	7.9×10^{-6}
rs11867936	17	2661224	*SMG6*	C/T	0.21	6.1×10^{-5}
rs117941260	17	4559946	*INCA1*	G/A	-0.25	8.7×10^{-5}
rs75069386	20	37854109	*DHX35-MAFB*	C/T	0.12	4.5×10^{-5}

Supplementary Table 3. Effect of the 47 lead IOP-associated SNPs on glaucoma (POAG case-control analysis)

				**GERA Meta-Analysis**			
**SNP**	**Chr**	**Locus**	**Alleles**	**OR**	***P***	***P*het**	***I*²**
rs1866758	1	*HIVEP3*	T/C	1.07	0.042	0.003	78.7
rs6668108	1	*LOC440700-TMCO1*	A/G	1.28	**2.8×10^{-8}**	0.81	0.0
rs596169	1	*MIR548F3*	A/G	0.91	0.12	0.80	0.0
rs115179432	2	*LTBP1*	A/G	0.92	0.18	0.17	44.3
rs7426380	2	*EFEMP1*	A/G	1.03	0.37	0.01	73.6
rs6732795	2	*ANTXR1*	A/C	0.96	0.22	0.83	0.0
rs55692468	2	*FMNL2*	T/G	0.91	0.0051	0.38	2.7
rs1035673	2	*TNS1*	T/C	0.93	0.02	0.38	1.6
rs143937055	2	*COL4A3/LOC654841*	TTTG/T	1.07	0.03	0.86	0.0
rs7599762	2	*COL6A3*	G/C	0.98	0.69	0.95	0.0
rs11710845	3	*LINC01214/TSC22D2*	C/T	1.07	0.06	0.89	0.0
rs7635832	3	*FNDC3B*	T/G	1.02	0.58	0.06	60.1
rs9853115	3	*DGKG-LINC02052*	T/A	1.07	0.04	0.03	66.4
rs13076750	3	*LPP*	A/G	1.02	0.48	0.89	0.0
rs28795989	4	*AFAP1*	A/G	1.16	3.6×10^{-6}	0.29	19.3
rs17527016	4	*PITX2-C4orf32*	C/T	1.13	0.0022	0.10	51.4
rs4865762	5	*MOCS2/FST*	C/A	1.03	0.33	0.41	0.0
rs73220188	5	*FBXL17/FER*	T/C	1.16	0.0037	0.43	0.0
rs2745572	6	*FOXF2-FOXCUT*	A/G	1.13	0.00029	0.30	18.7
rs1396046	6	*PKHD1*	G/A	0.94	0.03	0.63	0.0
rs2875087	6	*FAM46A-IBTK*	C/T	1.06	0.05	0.26	24.7
rs141917145	6	*near RFPL4B*	AAAGA AACAA G/A	1.07	0.04	0.26	24.4
rs1509922	7	*SEMA3C-HGF*	G/A	1.06	0.05	0.48	0.0
rs6968419	7	*TFEC-TES*	A/G	1.11	0.00081	0.42	0.0
rs10105844	8	*ANGPT1*	G/A	0.94	0.11	0.86	0.0
rs2224492	9	*GLIS3*	A/G	0.97	0.35	0.68	0.0
rs1831902	9	*FLJ41200-LINC00583*	C/T	1.02	0.62	0.40	0.0
rs2472493	9	*AK311445/ABCA1*	G/A	1.15	5.4×10^{-6}	0.46	0.0
rs11014632	10	*GPR158*	A/G	1.03	0.27	0.23	29.6
rs5785510	10	*ARID5B*	CA/C	1.04	0.27	0.91	0.0
rs66479974	10	*CYP26A1-MYOF*	CAG/C	0.99	0.83	0.12	49.4
rs79390637	11	*PSMC3/RAPSN*	C/A	1.08	0.019	0.03	66.0

rs199800298	11	*ARHGEF12*	AG/A	0.98	0.49	0.14	44.9
rs7977237	12	*LOC105369739-ADAMTS20*	T/A	1.04	0.22	0.13	47.6
rs74481774	12	*TMEM119*	A/G	1.14	0.24	0.99	0.0
rs9552680	13	*LINC00424-BASP1P1*	C/T	1.02	0.48	0.24	28.7
rs11616662	13	*FOXO1*	G/A	1.04	0.40	0.53	0.0
rs12912045	15	*SMAD3*	C/T	0.98	0.56	0.68	0.0
rs72755233	15	*ADAMTS17*	G/A	1.08	0.17	0.76	0.0
rs75828804	16	*ADAMTS18-NUDT7*	G/A	0.87	0.0078	0.09	53.2
rs12926024	16	*BANP-ZNF469*	T/C	0.99	0.87	0.88	0.0
rs4790881	17	*SMG6*	C/A	1.03	0.40	0.35	8.1
rs34629349	17	*INCA1*	C/A	1.06	0.06	0.22	31.5
rs9913911	17	*GAS7*	A/G	1.17	5.0×10^{-7}	0.21	33.1
rs11659764	18	*TCF4-LINC01415*	T/A	1.15	0.07	0.11	55.4
rs3918508	20	*LINC01734/LINC01370*	G/A	1.08	0.0096	0.30	17.9
rs2839082	21	*COL6A1-COL6A2*	C/T	1.04	0.19	0.82	0.0

OR, odd ratios; *P*het, heterogeneity *P*-values from Cochrane's Q statistic; I^2, heterogeneity index (0–100%); *P*-value in bold achieved genome-wide level of significance ($P < 5 \times 10^{-8}$); loci highlighted in grey have been previously identified in GWAS of IOP at a genome-wide level of significance.

Supplementary Table 4. Genetic association with related traits and eye diseases at the identified IOP loci

SNP[a]	Chr region	Locus	SNP[b]	Related traits and diseases	Ref	R^2	D'	Distance (kb) between SNP[a] and SNP[b]
rs6668108	1q24.1	*LOC440700-TMCO1*	rs4656461	POAG	[2]	0.99	1.0	4.1
rs6668108	1q24.1	*LOC440700-TMCO1*	rs7518099	POAG	[3]	1.0	1.0	45.6
rs7426380	2p16.1	*EFEMP1*	rs3791679	Cup area	[4]	0.33	0.98	1.9
rs7426380	2p16.1	*EFEMP1*	rs1346786	Cup area	[5]	0.49	0.98	13.3
rs7426380	2p16.1	*EFEMP1*	Rare mutation	Monogenic form of POAG in an African-American family	[6]	NA	NA	NA
rs6732795	2p16.1	*ANTXR1*	Rare mutations	GAPO syndrome (optic atrophy)	[7-9]	NA	NA	NA
rs143937055	2q36.3	*COL4A3/LOC654841*	rs7606754	CCT	[10]	0.99	1.0	8.8
rs7635832	3q26.3	*FNDC3B*	rs4894535	CCT and keratoconus	[10]	0.82	1.0	6.3
rs7635832	3q26.3	*FNDC3B*	rs6445055	POAG	[11]	0.92	1.0	3.1
rs7635832	3q26.3	*FNDC3B*	rs4894796	POAG	[12]	0.005	0.16	208.5
rs7635832	3q26.3	*FNDC3B*	rs16856870	PACG	[13]	0.03	0.32	194.8
rs28795989	4p16.1	*AFAP1*	rs4619890	POAG	[14]	0.25	0.57	38.4
rs28795989	4p16.1	*AFAP1*	rs11732100	POAG	[3]	0.51	0.97	33.1
rs17527016	4q25	*PITX2-C4orf32*	Rare mutations	Axenfeld-Rieger syndrome (anterior segment malformations)	[15,16]	NA	NA	NA
rs2745572	6p25.3	*FOXF2-FOXCUT (FOXC1)*	rs2745572	POAG	[3]	1.0	1.0	0.0
rs2745572	6p25.3	*FOXF2-FOXCUT (FOXC1)*	Rare mutations	Axenfeld-Rieger syndrome (anterior segment malformations)	[15,16]	NA	NA	NA
rs1396046	6p12.3	*PKHD1*	Rare mutation	Monogenic form of POAG in a Chinese family	[17]	NA	NA	NA
rs2875087	6q14.1	*FAM46A-IBTK*	rs1538138	CCT	[10,18]	0.0	0.11	178.5
rs6968419	7q31.2	*TFEC-TES (CAV1/CAV2)*	rs4236601	POAG	[19]	0.02	0.28	339.3
rs2224492	9p24.2	*GLIS3*	rs736893	PACG	[13]	0.71	0.85	20.5
rs2472493	9q31.1	*AK311445/ABCA1*	rs2472493	POAG	[3,14]	1.0	1.0	0.0
rs2472493	9q31.1	*AK311445/ABCA1*	rs2487032	POAG	[20]	0.43	0.78	8.1
rs5785510	10q21.2	*ARID5B*	rs7090871	CCT	[10]	0.98	1.0	7.0

rs199800298	11q23.3	*ARHGEF12 (/TMEM136)*	rs11827818	POAG, XFS	[14,21]	0.27	0.97	149.8
rs199800298	11q23.3	*ARHGEF12 (/TMEM136)*	rs58073046	POAG, HTG	[22]	0.18	0.99	100.1
rs11616662	13q14.1	*FOXO1*	rs2721051	CCT and keratoconus	[10]	0.99	1.0	8.6
rs12912045	15q22.3	*SMAD3*	rs12913547	CCT	[10]	0.99	0.99	0.2
rs12926024	16q24.2	*BANP-ZNF469*	rs9938149	CCT and keratoconus	[10]	0.97	0.99	0.3
rs9913911	17p13.1	*GAS7*	rs9897123	POAG	[3]	0.54	0.95	10.7
rs11659764	18q21.2	*TCF4-LINC01415*	Rare mutations; rs784257	Pitt-Hopkins syndrome (eye abnormalities); Fuchs endothelial corneal dystrophy	[23,24]	0.19	0.97	61.7

[a]index SNP associated with IOP in the current study; [b]index SNP associated with related traits and diseases in previous studies; SNPs highlighted in grey are identical between [a] and [b]; CCT, central corneal thickness; POAG, primary open-angle glaucoma; PACG, primary angle-closure glaucoma; XFS, exfoliation syndrome; HTG, high-tension glaucoma; linkage disequilibrium (LD) metrics (R^2 and D') have all been calculated in European-ancestry populations using either a web-based bioinformatic tool (https://analysistools.nci.nih.gov/LDlink/)[25] or PLINK[26] v1.9 (www.cog-genomics.org/plink/1.9/) software. As a note, SNP rs199800298 (deletion on chromosome 11) was not in 1000 Genomes reference panel; LD metrics between SNP rs199800298 and SNPs rs11827818 and r58073046 have been approximated using our non-Hispanic white GERA sample. rs numbers were not available (NA) for the rare missense mutations causing monogenic forms of POAG or other rare syndromes.

Supplementary Table 5. Expression of the genes in the 47 IOP loci that contained associated 95% credible set variants in adult human eye tissues

		The Ocular Tissue Database[1]											EyeSAGE[27,28]			
Chr	Gene	Probe ID	Choroid RPE	Ciliary Body	Cornea	Iris	Lens	Optic Nerve	Optic Nerve Head	Retina	Sclera	TM	TM	MAC	RPE MAC	RPE Peri
1	*HIVEP3*	2408681	25.0	32.2	27.7	29.2	24.2	34.7	27.4	29.4	29.2	27.8	+	+	-	-
1	*TMCO1*	2442134	205.4	138.5	224.6	138.8	149.5	143.4	205.3	145.4	119.1	188.4	+	NA	NA	NA
1	*MIR548F3*	NA	NA	NA	NA	NA	NA	NA	NA	NA	NA	NA	NA	NA	NA	NA
1	*LYPLAL1*	2380785	77.5	104.8	56.5	260.4	74.3	46.2	60.8	27.1	44.8	38.8	+	-	-	+
2	*LTBP1*	2476510	60.4	61.4	93.3	91.1	108.1	125.8	254.6	16.7	118.9	70.1	+	-	-	-
2	*EFEMP1*	2554018	562.2	667.8	165.3	324.4	219.3	352.7	363.1	39.3	332.0	214.3	+	+	+	+
2	*ANTXR1*	2487082	124.8	90.8	58.6	65.3	26.0	193.8	228.2	23.9	354.5	145.2	+	-	+	-
2	*GFPT1*	2558045	92.0	99.0	119.1	91.9	68.2	77.1	120.5	70.1	110.7	166.8	+	-	+	-
2	*NFU1*	2558118	76.6	89.0	46.8	137.1	130.0	41.6	67.5	52.0	32.1	56.4	+	NA	NA	NA
2	*AAK1*	2558150	41.5	45.0	44.7	32.6	82.9	48.3	64.7	134.9	36.0	74.3	+	+	+	-
2	*CACNB4*	2581349	25.6	21.8	25.8	24.7	19.4	34.7	36.4	30.5	39.7	38.0	NA	+	-	-
2	*STAM2*	2581430	105.5	93.0	112.5	86.9	184.0	127.1	175.9	122.0	95.0	131.7	+	NA	NA	NA
2	*FMNL2*	2510713	154.1	64.7	41.0	121.0	181.7	338.4	394.5	33.1	186.5	77.2	+	+	+	+
2	*TNS1*	2599153	34.8	47.2	29.5	35.2	25.1	49.9	58.4	26.7	43.1	136.7	+	NA	NA	NA
2	*COL4A3*	2530425	36.6	20.9	47.5	11.7	98.0	29.3	40.8	45.8	23.8	47.5	+	+	+	-
2	*LOC654841*	2602260	18.4	20.5	17.0	16.1	20.3	21.1	22.5	25.7	19.2	22.3	NA	NA	NA	NA
2	*MFF*	2530539	77.6	77.4	61.1	101.6	92.0	95.5	117.3	93.1	45.5	89.8	NA	NA	NA	NA
2	*COL6A3*	2605321	30.2	36.7	156.8	30.7	21.9	47.6	71.1	21.1	105.7	53.7	+	-	+	-
2	*MLPH*	2534252	48.1	39.7	47.5	43.7	36.5	36.1	40.0	35.3	39.5	39.5	+	-	-	-
2	*TRAF3IP1*	2534810	88.1	99.9	98.7	87.2	84.5	99.1	158.2	87.4	83.6	124.6	+	+	+	-
3	*FNDC3B*	2652410	73.4	60.6	80.0	44.1	90.9	46.5	75.2	47.2	112.6	69.8	+	-	-	+
3	*LPP*	2657250	92.9	94.2	56.0	62.2	51.2	68.2	107.4	28.8	81.7	213.2	+	+	+	+
4	*AFAP1*	2717518	46.3	34.2	60.5	44.5	50.7	59.2	60.2	46.4	43.8	59.6	NA	NA	NA	NA
4	*ELOVL6*	2781813	23.3	23.9	37.2	11.9	51.6	54.4	73.6	55.0	15.6	30.4	+	-	+	-
4	*ENPEP*	2739468	21.5	16.6	11.9	19.2	9.6	12.6	14.6	9.7	33.9	32.5	NA	NA	NA	NA
4	*PANCR*	NA	NA	NA	NA	NA	NA	NA	NA	NA	NA	NA	NA	NA	NA	NA
4	*PITX2*	2781914	45.0	51.9	41.4	39.6	25.4	38.6	30.6	26.9	47.0	53.7	+	NA	NA	NA
5	*FER*	2823326	91.8	64.8	101.4	77.2	78.6	104.9	150.0	90.4	94.3	108.8	+	-	-	-
6	*PKHD1*	2956904	17.0	17.9	17.2	16.5	15.1	16.3	17.7	19.1	17.3	13.5	NA	+	-	+
8	*ANGPT1*	3148463	193.8	20.8	51.9	7.0	11.7	168.2	148.4	15.0	114.7	78.3	+	-	+	+
8	*RSPO2*	3148545	35.9	20.0	8.1	27.7	8.5	26.6	26.5	11.4	14.7	35.9	+	NA	NA	NA

8	*EIF3E*	3148582	70.6	60.4	47.9	90.1	63.7	51.9	50.4	33.9	57.5	81.8	NA	NA	NA	NA
9	*GLIS3*	3160500	71.3	103.4	135.8	70.3	41.2	97.9	45.9	89.3	65.2	226.9	+	NA	NA	NA
10	*PRTFDC1*	3281703	40.9	25.1	14.0	34.0	11.7	102.1	83.4	51.9	19.3	29.2	NA	+	+	-
10	*GPR158*	3239437	17.2	17.6	15.5	13.6	16.0	62.4	96.9	29.6	15.5	17.4	NA	-	+	-
10	*ARID5B*	NA	NA	NA	NA	NA	NA	NA	NA	NA	NA	NA	+	+	+	-
10	*EXOC6*	3258260	29.3	20.7	62.2	9.1	38.3	24.6	25.2	55.0	18.0	32.9	+	NA	NA	NA
11	*PSMC3*	3372209	56.6	60.2	82.1	38.5	117.2	70.4	73.0	64.8	83.7	97.2	+	+	+	+
11	*RAPSN*	3372235	55.0	56.9	72.8	60.7	63.7	71.5	51.3	55.8	63.7	69.6	NA	NA	NA	NA
11	*TMEM136*	3352485	19.6	29.4	8.7	24.5	38.1	27.8	25.1	67.8	24.6	28.0	NA	NA	NA	NA
11	*ARHGEF12*	3352503	145.5	158.0	155.2	97.2	334.0	154.5	173.5	180.9	105.1	245.7	+	+	+	-
12	*ADAMTS20*	3451558	14.4	14.0	14.9	12.7	12.3	15.0	12.7	13.8	13.9	12.0	NA	-	-	+
12	*TMEM119*	3470503	31.7	27.4	32.3	26.9	31.6	41.5	39.2	34.3	36.9	32.4	+	NA	NA	NA
12	*SELPLG*	3470523	29.0	30.0	22.4	32.0	22.8	33.3	25.1	21.0	33.7	23.1	+	-	-	-
13	*FGF9*	3480885	55.9	27.2	88.4	28.6	7.4	24.9	29.5	212.0	16.7	93.0	NA	+	+	+
13	*LINC00424*	NA	NA	NA	NA	NA	NA	NA	NA	NA	NA	NA	NA	NA	NA	NA
13	*LINC00540*	NA	NA	NA	NA	NA	NA	NA	NA	NA	NA	NA	NA	NA	NA	NA
13	*LINC00621*	NA	NA	NA	NA	NA	NA	NA	NA	NA	NA	NA	NA	NA	NA	NA
13	*SGCG*	3481296	23.3	36.7	20.6	24.3	9.8	23.7	14.3	14.3	20.9	58.3	NA	NA	NA	NA
13	*SACS*	3505319	17.8	16.1	10.7	13.1	38.1	34.8	22.9	15.8	13.4	25.4	+	+	+	+
13	*LINC00327*	NA	NA	NA	NA	NA	NA	NA	NA	NA	NA	NA	NA	NA	NA	NA
13	*TNFRSF19*	3481410	35.5	36.1	31.5	29.7	35.3	39.7	36.8	31.6	35.3	33.4	+	+	-	-
13	*FOXO1*	3510858	90.9	92.7	138.9	84.5	123.6	124.0	110.9	92.3	149.7	160.4	+	-	+	-
15	*SMAD3*	3598959	141.7	117.9	222.9	92.7	82.1	77.9	78.7	43.9	111.4	104.5	+	-	-	-
15	*ADAMTS17*	3641633	50.6	42.6	41.3	36.2	41.6	45.2	45.7	44.6	57.4	46.7	+	-	-	+
17	*SMG6*	3740838	27.5	25.8	35.6	22.9	44.0	34.6	33.5	33.5	34.1	38.9	+	NA	NA	NA
17	*SLC52A1*	3742532	27.9	32.4	36.4	29.2	38.0	30.0	31.7	38.0	42.6	38.9	NA	NA	NA	NA
17	*GAS7*	3744965	100.6	63.2	19.7	84.8	188.1	219.1	230.5	102.9	77.2	128.0	NA	+	+	+
18	*TCF4*	3808854	122.6	57.5	104.7	85.9	51.8	97.5	145.2	85.3	243.0	229.7	+	+	-	-
	All	mean	73.1	65.5	64.5	60.0	67.1	77.7	90.2	54.3	72.6	82.5				
		SD	82.9	91.9	52.7	58.7	64.2	71.8	85.1	43.3	72.4	63.7				

In the Ocular Tissue Database[1] (OTDB), the gene expression is indicated as Affymetrix Probe Logarithmic Intensity Error (PLIER) number. The PLIER numbers were calculated by GC-background correction, PLIER normalization, log transformation and *z*-score calculation. The OTDB is also available at https://genome.uiowa.edu/otdb/.
In the EyeSAGE[27,28] datasets from NEIBank, the gene expression is determined by tag counts in the Serial Analysis of Gene Expression (SAGE). All counts were summarized for each gene per tissue, and we added '+' to indicate the expression, or '-' to label the no expression while the counts are 0. The EyeSAGE is publicly available at the http://neibank.nei.nih.gov/EyeSAGE/index.shtml.
Abbreviations: RPE, retinal pigment epithelium; TM, trabecular meshwork; MAC, retina macular; RPE Peri, RPE peripheral; na, not applicable.

Supplementary Table 6. Top gene prioritization according to DEPICT including our 47 lead SNPs (P < 5x10^{-8} in the discovery GERA cohort). Nominal associations are reported.

Locus	N	Chr - Position	Gene	Nominal *P*-value	FDR<5%	Top cis eQTL SNP (Westra *et al*. Nature Genetics 2013)
rs2839082	1	chr21:47401651-47424964	*COL6A1*	0.0084	No	rs2839029
rs11616662	1	chr13:41129817-41240734	*FOXO1*	0.0096	No	-
rs2224492	1	chr9:3824127-4348392	*GLIS3*	0.0116	No	rs10758547;rs806053;rs10974485
rs7426380	1	chr2:56093102-56151274	*EFEMP1*	0.0139	No	rs6722720
rs6968419	4	chr7:115575202-116148595	*CAV2*	0.0290	No	rs6466574;rs16869914;rs12672038
rs6732795	1	chr2:69240310-69476459	*ANTXR1*	0.0330	No	-
rs13076750	1	chr3:187871072-188608460	*LPP*	0.0419	No	rs6659

N, number of genes in locus; FDR, false discovery rate

Supplementary Table 7. Gene set enrichment analysis in DEPICT using the 47 lead SNPs ($P < 5x10^{-8}$ in the discovery GERA cohort). Top 20 pathways are shown for IOP identified loci.

Original gene set ID	Original gene set description	Nominal *P*-value	FDR < 5%
MP:0006055	abnormal vascular endothelial cell morphology	**6.2×10^{-9}**	**Yes**
MP:0000284	double outlet heart right ventricle	2.1×10^{-5}	No
MP:0010402	ventricular septal defect	4.8×10^{-5}	No
ENSG00000100311	PDGFB subnetwork	1.0×10^{-4}	No
MP:0000010	abnormal abdominal fat pad morphology	1.1×10^{-4}	No
ENSG00000185722	ANKFY1 subnetwork	1.2×10^{-4}	No
ENSG00000184895	SRY subnetwork	1.3×10^{-4}	No
GO:0032350	regulation of hormone metabolic process	1.4×10^{-4}	No
MP:0008028	pregnancy-related premature death	2.1×10^{-4}	No
ENSG00000143569	UBAP2L subnetwork	2.3×10^{-4}	No
MP:0006027	impaired lung alveolus development	2.6×10^{-4}	No
GO:0006700	C21-steroid hormone biosynthetic process	2.7×10^{-4}	No
GO:0008212	mineralocorticoid metabolic process	3.7×10^{-4}	No
ENSG00000174827	PDZK1 subnetwork	3.7×10^{-4}	No
MP:0009743	preaxial polydactyly	4.4×10^{-4}	No
MP:0002747	abnormal aortic valve morphology	5.0×10^{-4}	No
MP:0011090	partial perinatal lethality	5.4×10^{-4}	No
ENSG00000161570	CCL5 subnetwork	5.5×10^{-4}	No
ENSG00000203283	ENSG00000203283 subnetwork	5.5×10^{-4}	No
ENSG00000113070	HBEGF subnetwork	6.4×10^{-4}	No

FDR, false discovery rate

Supplementary Table 8. Top results tissue enrichment in DEPICT, using our 47 lead SNPs ($P < 5x10^{-8}$ in the discovery GERA cohort). Nominal associations are reported.

MeSH term	MeSH first level term	MeSH second level term	Nominal *P*-value	FDR<5%
A15.145.846	Serum	Hemic and Immune Systems	0.0069	No
A10.165.450.300	Cicatrix	Tissues	0.0229	No
A10.165.450	Granulation Tissue	Tissues	0.0229	No
A10.690.467	Muscle Smooth	Tissues	0.0284	No
A03.556.875	Upper Gastrointestinal Tract	Digestive System	0.0305	No
A03.556.875.875	Stomach	Digestive System	0.0307	No
A11.329.114	Adipocytes	Cells	0.0312	No
A07.231.114	Arteries	Cardiovascular System	0.0385	No
A11.620	Muscle Cells	Cells	0.0423	No
A11.620.520	Myocytes Smooth Muscle	Cells	0.0423	No
A05.360.319.679.690	Myometrium	Urogenital System	0.0497	No

FDR, false discovery rate

Supplementary Table 9. Replication of SNP associations reported in previous IOP GWAS studies in the GERA cohort

					Meta-analysis				NHW		H/L		EAS		AA	
**Chr**	**SNP**	**Nearest Gene**	**Ref**	**Al**	**β**	***P***	***P*het**	***I^2***	**β**	***P***	**β**	***P***	**β**	***P***	**β**	***P***
1	rs7555523	*TMCO1*	11,29	C/A	0.21	**1.5×10^{-18}**	0.02	70.2	0.24	**3.0×10^{-20}**	0.10	0.20	-0.09	0.67	-0.10	0.50
3	rs6445055	*FNDC3B*	11	G/A	0.23	**1.5×10^{-32}**	0.87	0.0	0.24	**1.1×10^{-25}**	0.23	0.0002	0.23	5.3×10^{-5}	0.15	0.14
7	rs59072263	*GLCCI1/ICA1*	30	G/T	0.01	0.54	0.45	0.0	-0.0014	0.95	0.11	0.17	0.04	0.70	0.12	0.32
7	rs10258482	*CAV1*	11	C/A	-0.13	**2.1×10^{-14}**	0.29	19.9	-0.13	**4.6×10^{-13}**	-0.18	0.0034	-0.14	0.39	0.05	0.63
7	rs4236601	*CAV1*	20	G/A	-0.13	**1.5×10^{-14}**	0.29	19.4	-0.13	**4.3×10^{-13}**	-0.18	0.0024	-0.13	0.42	0.04	0.68
9	rs2472493	*ABCA1*	11	G/A	0.17	**1.1×10^{-28}**	0.37	4.9	0.18	**1.3×10^{-26}**	0.10	0.05	0.12	0.023	0.17	0.07
9	rs2286885	*MVB12B (FAM125B)*	31	A/C	0.05	0.0013	0.37	5.5	0.04	0.0086	0.11	0.0307	0.06	0.26	-0.08	0.43
9	rs8176693	*ABO*	11	C/T	-0.14	2.6×10^{-7}	0.67	0.0	-0.12	0.0001	-0.15	0.15	-0.20	0.0036	-0.24	0.11
11	rs747782	*NUP160-PTPRJ*	11	T/C	-0.10	2.1×10^{-7}	0.19	37.3	-0.08	0.0003	-0.19	0.0074	-0.18	0.0023	-0.15	0.12
11	rs58073046	*ARHGEF12*	22	A/G	-0.19	**6.2×10^{-15}**	0.93	0.0	-0.19	**8.0×10^{-13}**	-0.15	0.14	-0.19	0.0049	-0.06	0.80
11	rs55796939	*ADAMTS8*	4	C/T	-0.20	3.5×10^{-6}	0.39	0.9	-0.18	0.0003	-0.45	0.0062	-0.15	0.12	-0.35	0.18
17	rs9913911	*GAS7*	11	A/G	0.21	**1.4×10^{-42}**	0.20	35.4	0.22	**5.5×10^{-38}**	0.10	0.06	0.23	6.0×10^{-6}	0.17	0.14
17	rs11656696	*GAS7*	29	C/A	0.17	**9.9×10^{-29}**	0.78	0.0	0.18	**1.3×10^{-24}**	0.12	0.029	0.19	0.0003	0.16	0.15

NHW: non-Hispanic whites; H/L: Hispanic/Latinos; EAS: East Asians; AA: African Americans; Al, alleles; *P*het, heterogeneity *P*-values from Cochrane's Q statistic; I^2, heterogeneity index (0–100%); *P*-values in bold achieved genome-wide level of significance ($P < 5 \times 10^{-8}$)

Supplementary Table 10. Proportion of IOP variance explained by lead variants newly- or previously-identified

GERA cohort	47 lead SNPs identified in the current study		13 SNPs previously identified
Race/ethnicity	Adjusted R^2 (%) Multiple IOP measurements	Adjusted R^2 (%) Single IOP measurement	Adjusted R^2 (%) Multiple IOP measurements
**NHW**	3.66	2.88	1.09
**H/L**	3.01	2.60	0.84
**EAS**	3.54	2.58	1.17
**AA**	2.80	2.65	0.25

NHW: non-Hispanic whites; H/L: Hispanic/Latinos; EAS: East Asians; AA: African-Americans

Supplementary Table 11. Methods used to measure IOP in GERA cohort

IOP Measurement Methods	Percent (%)
Goldmann applanation tonometer	90.6
non-contact tonometer	7.8
Tono-Pen XL	1.3
iCare rebound tonometer	0.1
Pneumotonometer / other	0.2

Supplementary Table 12. GERA covariate association with age at the median of IOP measurement, sex, and the ancestry PCs. For the interpretation of the PCs, see Banda *et al.* Genetics 2015[32]. Effect size estimates (β) and p-values are from the multivariate regression model.

**Non-Hispanic whites**			
	**β**	**SE**	***P*-value**
Age at the median IOP	-0.007	0.001	2.7×10^{-12}
Sex	0.34	0.02	1.4×10^{-48}
PC1	-0.26	1.56	0.97
PC2	4.13	1.74	0.02
PC3	-3.63	1.97	0.06
PC4	5.47	2.03	0.007
PC5	2.45	2.47	0.32
PC6	-2.72	2.05	0.18
PC7	-0.38	2.25	0.86
PC8	0.47	2.19	0.83
PC9	-1.18	2.14	0.58
PC10	4.08	2.15	0.06
ASHK	-0.55	0.07	1.2×10^{-14}
**Hispanic/Latinos**			
	**β**	**SE**	***P*-value**
Age at the median IOP	0.0007	0.003	0.78
Sex	0.33	0.07	1.1×10^{-5}
PC1	-2.13	4.26	0.62
PC2	6.76	3.63	0.06
PC3	-13.56	3.38	6.1×10^{-5}
PC4	2.73	3.89	0.48
PC5	0.56	3.31	0.86
PC6	3.59	3.36	0.28
**East Asians**			
	**β**	**SE**	***P*-value**
Age at the median IOP	0.003	0.003	0.25
Sex	0.037	0.074	0.61
PC1	-11.45	3.27	4.7×10^{-4}
PC2	13.15	3.05	1.6×10^{-5}
PC3	10.15	3.06	9.1×10^{-4}
PC4	-0.98	3.08	0.75
PC5	-4.38	3.12	0.16
PC6	2.72	3.11	0.38
**African Americans**			
	**β**	**SE**	***P*-value**
Age at the median IOP	0.01	0.005	0.004
Sex	0.16	0.14	0.24
PC1	-4.06	3.65	0.27
PC2	2.23	3.68	0.54
PC3	4.46	3.66	0.22
PC4	1.20	3.66	0.74
PC5	11.07	3.64	0.002
PC6	11.29	3.61	0.002

Abbreviations: PC, principal component; ASHK, Ashkenazi; SE, standard error

REFERENCES

1 Wagner, A. H. *et al.* Exon-level expression profiling of ocular tissues. *Exp Eye Res* **111**, 105-111, doi:10.1016/j.exer.2013.03.004 (2013).

2 Burdon, K. P. *et al.* Genome-wide association study identifies susceptibility loci for open angle glaucoma at TMCO1 and CDKN2B-AS1. *Nat Genet* **43**, 574-578, doi:10.1038/ng.824 (2011).

3 Bailey, J. N. *et al.* Genome-wide association analysis identifies TXNRD2, ATXN2 and FOXC1 as susceptibility loci for primary open-angle glaucoma. *Nat Genet* **48**, 189-194, doi:10.1038/ng.3482 (2016).

4 Springelkamp, H. *et al.* New insights into the genetics of primary open-angle glaucoma based on meta-analyses of intraocular pressure and optic disc characteristics. *Hum Mol Genet*, doi:10.1093/hmg/ddw399 (2017).

5 Springelkamp, H. *et al.* Meta-analysis of Genome-Wide Association Studies Identifies Novel Loci Associated With Optic Disc Morphology. *Genet Epidemiol* **39**, 207-216, doi:10.1002/gepi.21886 (2015).

6 Mackay, D. S., Bennett, T. M. & Shiels, A. Exome Sequencing Identifies a Missense Variant in EFEMP1 Co-Segregating in a Family with Autosomal Dominant Primary Open-Angle Glaucoma. *PLoS One* **10**, e0132529, doi:10.1371/journal.pone.0132529 (2015).

7 Bayram, Y. *et al.* Whole exome sequencing identifies three novel mutations in ANTXR1 in families with GAPO syndrome. *Am J Med Genet A* **164A**, 2328-2334, doi:10.1002/ajmg.a.36678 (2014).

8 Salas-Alanis, J. C. *et al.* New ANTXR1 Gene Mutation for GAPO Syndrome: A Case Report. *Mol Syndromol* **7**, 160-163, doi:10.1159/000446619 (2016).

9 Stranecky, V. *et al.* Mutations in ANTXR1 cause GAPO syndrome. *Am J Hum Genet* **92**, 792-799, doi:10.1016/j.ajhg.2013.03.023 (2013).

10 Lu, Y. *et al.* Genome-wide association analyses identify multiple loci associated with central corneal thickness and keratoconus. *Nat Genet* **45**, 155-163, doi:10.1038/ng.2506 (2013).

11 Hysi, P. G. *et al.* Genome-wide analysis of multi-ancestry cohorts identifies new loci influencing intraocular pressure and susceptibility to glaucoma. *Nat Genet* **46**, 1126-1130, doi:10.1038/ng.3087 (2014).

12 Li, Z. *et al.* A common variant near TGFBR3 is associated with primary open angle glaucoma. *Hum Mol Genet* **24**, 3880-3892, doi:10.1093/hmg/ddv128 (2015).

13 Khor, C. C. *et al.* Genome-wide association study identifies five new susceptibility loci for primary angle closure glaucoma. *Nat Genet* **48**, 556-562, doi:10.1038/ng.3540 (2016).

14 Gharahkhani, P. *et al.* Common variants near ABCA1, AFAP1 and GMDS confer risk of primary open-angle glaucoma. *Nat Genet* **46**, 1120-1125, doi:10.1038/ng.3079 (2014).

15 Lewis, C. *et al.* Primary Congenital and Developmental Glaucomas. *Hum Mol Genet*, doi:10.1093/hmg/ddx205 (2017).

16 Souzeau, E. *et al.* Glaucoma spectrum and age-related prevalence of individuals with FOXC1 and PITX2 variants. *Eur J Hum Genet* **25**, 839-847, doi:10.1038/ejhg.2017.59 (2017).

17 Liu, T., Xie, L., Ye, J. & He, X. Family-based analysis identified CD2 as a susceptibility gene for primary open angle glaucoma in Chinese Han population. *J Cell Mol Med* **18**, 600-609, doi:10.1111/jcmm.12201 (2014).

18 Cornes, B. K. *et al.* Identification of four novel variants that influence central corneal thickness in multi-ethnic Asian populations. *Hum Mol Genet* **21**, 437-445, doi:10.1093/hmg/ddr463 (2012).

19 Thorleifsson, G. *et al.* Common variants near CAV1 and CAV2 are associated with primary open-angle glaucoma. *Nat Genet* **42**, 906-909, doi:10.1038/ng.661 (2010).

20 Chen, F. *et al.* Exome array analysis identifies CAV1/CAV2 as a susceptibility locus for intraocular pressure. *Invest Ophthalmol Vis Sci* **56**, 544-551, doi:10.1167/iovs.14-15204 (2014).
21 Aung, T. *et al.* Genetic association study of exfoliation syndrome identifies a protective rare variant at LOXL1 and five new susceptibility loci. *Nat Genet* **49**, 993-1004, doi:10.1038/ng.3875 (2017).
22 Springelkamp, H. *et al.* ARHGEF12 influences the risk of glaucoma by increasing intraocular pressure. *Hum Mol Genet* **24**, 2689-2699, doi:10.1093/hmg/ddv027 (2015).
23 Afshari, N. A. *et al.* Genome-wide association study identifies three novel loci in Fuchs endothelial corneal dystrophy. *Nat Commun* **8**, 14898, doi:10.1038/ncomms14898 (2017).
24 Marangi, G. & Zollino, M. Pitt-Hopkins Syndrome and Differential Diagnosis: A Molecular and Clinical Challenge. *J Pediatr Genet* **4**, 168-176, doi:10.1055/s-0035-1564570 (2015).
25 Machiela, M. J. & Chanock, S. J. LDlink: a web-based application for exploring population-specific haplotype structure and linking correlated alleles of possible functional variants. *Bioinformatics* **31**, 3555-3557, doi:10.1093/bioinformatics/btv402 (2015).
26 Chang, C. C. *et al.* Second-generation PLINK: rising to the challenge of larger and richer datasets. *Gigascience* **4**, 7, doi:10.1186/s13742-015-0047-8 (2015).
27 Bowes Rickman, C. *et al.* Defining the human macula transcriptome and candidate retinal disease genes using EyeSAGE. *Invest Ophthalmol Vis Sci* **47**, 2305-2316, doi:10.1167/iovs.05-1437 (2006).
28 Liu, Y. *et al.* Serial analysis of gene expression (SAGE) in normal human trabecular meshwork. *Mol Vis* **17**, 885-893 (2011).
29 van Koolwijk, L. M. *et al.* Common genetic determinants of intraocular pressure and primary open-angle glaucoma. *PLoS Genet* **8**, e1002611, doi:10.1371/journal.pgen.1002611 (2012).
30 Genome-wide association study of intraocular pressure identifies the GLCCI1/ICA1 region as a glaucoma susceptibility locus. *Hum Mol Genet* **22**, 4653-4660, doi:10.1093/hmg/ddt293 (2013).
31 Nag, A. *et al.* A genome-wide association study of intra-ocular pressure suggests a novel association in the gene FAM125B in the TwinsUK cohort. *Hum Mol Genet* **23**, 3343-3348, doi:10.1093/hmg/ddu050 (2014).
32 Banda, Y. *et al.* Characterizing Race/Ethnicity and Genetic Ancestry for 100,000 Subjects in the Genetic Epidemiology Research on Adult Health and Aging (GERA) Cohort. *Genetics* **200**, 1285-1295, doi:10.1534/genetics.115.178616 (2015).
