# Supplementary material for: A large multi-ethnic genome-wide association study identifies novel genetic loci for intraocular pressure
Source: Nat Commun. 2017 Dec 13;8:2108. doi: 10.1038/s41467-017-01913-6 (PMC5727399; doi:10.1038/s41467-017-01913-6)
Supplement: Supplementary file 2 — Description of Additional Supplementary Files [file 41467_2017_1913_MOESM2_ESM.pdf]

Description of Additional Supplementary Files

File Name: Supplementary Data 1
Description: Supplementary Data 1. List of the 95% credible set of variants in each of the 47 IOP loci.
