# Supplementary material for: A large multi-ethnic genome-wide association study identifies novel genetic loci for intraocular pressure
Source: Nat Commun. 2017 Dec 13;8:2108. doi: 10.1038/s41467-017-01913-6 (PMC5727399; doi:10.1038/s41467-017-01913-6)
Supplement: Supplementary file 4 — Peer Review File [file 41467_2017_1913_MOESM4_ESM.pdf]

Reviewer #1 (Remarks to the Author):

Helene Choquet, Eric Jorgenson, and colleagues present a meta-analysis for intraocular pressure (IOP) in a large, multi-ethnic sample with electronic health records comprising 60,032 non-Hispanic individuals of European descent, 5,790 individuals of Hispanic/Latino descent, 5,215 individuals of East Asian descent, and 2,070 individuals of African descent. They replicated genome-wide significant results in a further 37,930 individuals from a previously published study where summary statistics were available for IOP. The authors identified 40 new genetic loci strongly influencing the IOP trait.

This study is remarkable in several respects. Firstly, each person had, on average, a total of 5.1 IOP measurements, and furthermore, the mean IOP of both eyes was calculated and the median of the mean IOP of both eyes were used as the response variable. Their analysis revalidated previously published loci at high levels of statistical significance (e.g. at TMCO1, FNDC3B, ABCA1, GAS7, and others), apart from identifying new loci. This is, to date, the most definitive study of genetic control of IOP to date. The statistical analysis is very well done, with appropriate quality control measures and corrections undertaken (e.g. age, gender, and PCs) to ensure veracity of the results. This reviewer agrees with the authors that the genomic inflation factor of 1.12 is in keeping with grossly polygenic traits (see Hyde CL et al., Nature Genetics 2016 for depression, Okbay A et al., Nature 2016 for education attainment, etc) and is not suggestive of population stratification in this large study.

I have a few suggestions for the authors to enhance the manuscript in view of aiding the readers prior to publication.

1. How many out of the 73,107 participants are diagnosed with glaucoma clinically? This could potentially be obtained from the electronic medical records. Do any of the 47 genome-wide significant loci show directionally consistent association when analyzed for POAG vs controls in GERA (this study)?

2. How many of the patients' IOP measurements were obtained post treatment? (although it would not significantly impact the robust genetic findings, this piece of information would be very helpful to the readers, especially glaucoma specialists and ophthalmologists).

3. It may be helpful to present quantile-quantile plots and genomic inflation factor separately for each of the following collections: 60,032 non-Hispanic individuals of European descent, 5,790 individuals of Hispanic/Latino descent, 5,215 individuals of East Asian descent, and 2,070 individuals of African descent, to accompany the overall meta-analysis QQ plot currently shown in Supplementary Figure 1.

4. Please include measures of heterogeneity for the meta-analysis (Phet, derived from Cochran's Q test and I2 index of heterogeneity) shown in Supplementary Table 1 and Supplementary Table 7.

5. At first glance, many of the 47 loci overlap in terms of locus annotation with other eye diseases such as glaucoma and age-related macular degeneration. Checking through the list of loci and SNPs, could the authors provide linkage disequilibrium metrices (r2 and D') between their index SNPs and the SNPs previously reported to be associated with the other eye diseases?
For example, SNP rs28795989 at AFAP1 was reported in Table 2 by the authors to associate with IOP, whereas SNP AFAP1 rs4619890 was reported by Gharahkhani P et al., [Nature Genetics 2014] to be associated with increased POAG risk. In addition, SNP rs11732100, also at AFAP1, was reported to associate with increased POAG risk by Bailey JN et al., (reference #27 in the manuscript text). What is the relationship between the authors' top SNP rs28795989 with the other two SNPs (rs4619890 and rs11732100), for example? Filling in this detail would enhance the manuscript further by aiding the broad readership of the journal.

6. Whilst scrutinizing Table 2, I note that SNP rs199800298 (ARHGEF12) could be linked to rs11827818, which was highlighted by Gharahkhani P et al., [Nature Genetics 2014]. This same marker has also been recently reported to associate with pseudo-exfoliation syndrome and glaucoma (Aung T et al., Nature Genetics 2017). As the authors emphasized a 'shared genetic etiology between glaucoma and related traits' in line 179 of the discussion section, some further analysis at this section could also aid in the overall relationship between genetic alleles and shared pleiotropy between the potentially related traits (e.g. pseudo-exfoliation syndrome is the commonest cause of POAG).

7. Supplementary Table 3 examined expression of the 47 genes or genes nearest to the index SNPs reported in this meta-analysis. Whilst this is helpful in the current manuscript, data in Table 2 showed that each locus was actually underlined by more than 1 gene, and sometimes, as many as 5-6 genes could be mapping to the 95% credible set underlined by the index SNP. In this light, could the authors
a) identify the total number of genes defined by the 95% credible set at each locus (this should be more than 47).
b) annotate them for expression as per the Ocular Tissue Database and EyeSage, as the authors have done for the 47 closest genes.
Doing so would be in keeping with Fritsche LG et al., (reference #81 in their manuscript text), which attempts to provide a more complete understanding of the genes and pathways implicated by the meta-analysis. Performing the DEPICT analysis (Supplementary Table 4) on a larger gene set implicated by the 95% credible set could uncover further biological insights implicated by the loci.

Reviewer #2 (Remarks to the Author):

This manuscript reports a GWAS to search for factors that influence IOP in a remarkable analysis of 373,967 measurements from 73,2017 subjects identified through electronic medical record. A total of 47 new loci for IOP factors were identified, 40 of which were novel. This report makes a major contribution to the study of the genetic basis increasing the number of factors. The amount of IOP variability explained by known genetic factors was increased by 3-fold, but still accounts for a tiny fraction of the phenotype (3%).

This major analysis reports a valuable contribution by identifying many novel genetic factors that influence IOP.

I have one significant concern.

The authors report identifying 47 genome-wide significant loci, 7 of which were previously reported. Of the 40 novel loci, only 14 met multiple measures threshold for significance in a second cohort ($p = 0.05/40 = 0.0013$). Suggestive p-values should not be evidence of confirmation. The authors should report throughout their report (title, abstract, results, discussion) that they discovered 14 novel loci (not 40). Replication is a key step in validating GWAS findings. The authors should clarify that the remaining 26 loci failed to replicate – i.e. they cannot confirm the association. It would be helpful to focus the discussion on the strongly replicated loci (i.e. FMNL2)

It is unclear why calculations for proportion of IOP variance; the DEPICT analyses; etc were calculated for loci / SNPs that did not replicate in a second cohort. What is the rationale for analyzing data that did not meet the threshold for statistical significance? Why not do this analysis with the confirmed loci?

Page 10, PKHD1, PITX2 EFEMP1 were not confirmed as an IOP loci with the second cohort. The

failure to replicate should be more clear in discussion of these (and other) loci. The discussion that milder mutations in genes that cause monogenic forms of glaucoma interesting, however, the specific examples are not well supported or described (the prior PlosOne report of a rare mutation in EFEMP1 in a single POAG family is suggestive but unconfirmed. The reference for PKHD1 is similarly suggestive).

Other minor concerns

Page 3- Line 52. The authors mention two features that might alter IOP variability within one of a patient's eyes. It would be helpful for them to begin by describing the known contributors to variability in IOP overall: time of day at which the measurement was taken (mentioned), corneal thickness, medications, and other demographic factors (age / race / etc). Also, the order at which measurements are made (between left and right eye) is a small, clinically insignificant difference, as their reference notes. Multiple different measurement techniques were employed (i.e. Goldmann tonometer, Tonopen XL, or others) and are another source of variability.

Page 5, line 84. It would be helpful to define lambda (genomic inflation)

Page 10, line 189. Mutations in FOXC1 and PITX2 have been shown to cause autosomal dominant Axenfeld-Rieger Syndrome, which has abnormal iridocorneal angle structures as a key feature. It would be helpful to refer to these autosomal dominant (Mendelian conditions) as Axenfeld-Rieger Syndrome.

Page 10, line 201-3. The authors should be more precise in their description of the anatomy of the outflow pathway – throughout the manuscript but here in particular. The trabecular meshwork is comprised of three layers (one of which is the juxtacanalicular layer, which is not separate from the TM as the sentence suggests).

Page 15, 319. It would be helpful to readers (especially for online methods without space limitations) to provide the QC methods and outcomes for this large study rather than a reference.

Reviewers' comments:

Reviewer #1 (Remarks to the Author):

Helene Choquet, Eric Jorgenson, and colleagues present a meta-analysis for intraocular pressure (IOP) in a large, multi-ethnic sample with electronic health records comprising 60,032 non-Hispanic individuals of European descent, 5,790 individuals of Hispanic/Latino descent, 5,215 individuals of East Asian descent, and 2,070 individuals of African descent. They replicated genome-wide significant results in a further 37,930 individuals from a previously published study where summary statistics were available for IOP. The authors identified 40 new genetic loci strongly influencing the IOP trait.

This study is remarkable in several respects. Firstly, each person had, on average, a total of 5.1 IOP measurements, and furthermore, the mean IOP of both eyes was calculated and the median of the mean IOP of both eyes were used as the response variable. Their analysis revalidated previously published loci at high levels of statistical significance (e.g. at TMCO1, FNDC3B, ABCA1, GAS7, and others), apart from identifying new loci. This is, to date, the most definitive study of genetic control of IOP to date. The statistical analysis is very well done, with appropriate quality control measures and corrections undertaken (e.g. age, gender, and PCs) to ensure veracity of the results. This reviewer agrees with the authors that the genomic inflation factor of 1.12 is in keeping with grossly polygenic traits (see Hyde CL et al., Nature Genetics 2016 for depression, Okbay A et al., Nature 2016 for education attainment, etc) and is not suggestive of population stratification in this large study.

I have a few suggestions for the authors to enhance the manuscript in view of aiding the readers prior to publication.

We thank the reviewer for the very supportive comments, and for the excellent suggestions.

1. **How many out of the 73,107 participants are diagnosed with glaucoma clinically? This could potentially be obtained from the electronic medical records. Do any of the 47 genome-wide significant loci show directionally consistent association when analyzed for POAG vs controls in GERA (this study)?**

This is an important point highlighted by the Reviewer. Among participants included in the current IOP study, 2,338 have been diagnosed with “glaucoma” clinically. We defined “glaucoma” as having at least two diagnoses of primary-open angle glaucoma (POAG), or two diagnoses of normal tension glaucoma (NTG), or one diagnosis of POAG and one diagnosis of NTG. In all cases, at least one of the diagnoses was made by a Kaiser Permanente ophthalmologist. To investigate whether our 47 lead IOP-associated SNPs were also associated with glaucoma, we performed a case-control analysis. For the control group, subjects who had one or more diagnosis of any type of glaucoma (e.g. pseudoexfoliation, pigmentary, or PACG) were excluded. Forty-two of the 47 genome-wide significant IOP-loci (89.4%) show directionally consistent association with POAG. We have now added a Supplementary Table 3 reporting these results, and added some text in the manuscript to reflect this point, as below:

In the Results section:

“***Effect of the 47 IOP-associated loci on glaucoma***

To investigate whether the 47 lead IOP-associated SNPs were also associated with primary open-angle glaucoma (POAG) susceptibility, we performed a case-control analysis in GERA including 2,338 POAG cases (normal and high-tension glaucoma) and 58,172 controls (Table 1). Forty-two of the 47 genome-

wide significant IOP-loci (89.4%) show directionally consistent association with POAG. In particular, we found associations with glaucoma at a Bonferroni level of significance (*P*<0.00106=0.05/47) for 6 SNPs. This included one SNP at a genome-wide level of significance for a previously identified locus at *TMCO1* (P=2.8×10^{-8} for rs6668108) (Supplementary Table 3). … Other Bonferroni significant loci included *AFAP1* (P=3.6×10^{-6} for rs28795989), *ABCA1* (P=5.4×10^{-6} for rs2472493), and *GAS7* (P=5.0×10^{-7} for rs9913911) (Supplementary Table 3). … We also found supportive evidence for the previously identified locus on *FOXC1* (P=2.9×10^{-4}) (Supplementary Table 3) with the same strongest SNP rs2745572 reported by Bailey et al. (Supplementary Table 4). All above-mentioned alleles associated with higher IOP levels also raised glaucoma risk (Supplementary Table 3). Finally, we identified nominal associations (0.00106 < P ≤ 0.05) with glaucoma for an additional 14 IOP-associated loci identified in the current study (Supplementary Table 3)."

In the Methods section:

"***Glaucoma Cases and Controls***
Among the 69,756 participants included in the current IOP study, 2,338 have been diagnosed with "glaucoma" clinically (Table 1). We defined "glaucoma" as having at least: two diagnoses of primary-open angle glaucoma (POAG), or two diagnoses of normal tension glaucoma (NTG), or one diagnosis of POAG and one diagnosis of NTG. In all cases, at least one of the diagnoses was made by a Kaiser Permanente ophthalmologist. For the control group, participants who had one or more diagnosis of any type of glaucoma (e.g. pseudoexfoliation, pigmentary, or PACG) were excluded. The final control sample included 58,172 participants.

Glaucoma Case-Control Analysis
We evaluated the associations of the 47 lead IOP-associated SNPs with glaucoma susceptibility by logistic regression under an additive model, and adjusting for age, sex and ancestry PCs."

2. **How many of the patients' IOP measurements were obtained post treatment? (although it would not significantly impact the robust genetic findings, this piece of information would be very helpful to the readers, especially glaucoma specialists and ophthalmologists).**

For this study, 116,980 IOP measurements from 3,632 participants were obtained after prescription of IOP lowering medications, however, in our analysis, we had removed those IOP measurements that were taken post-treatment. We now provide this number in the Methods section, as follows:

"For this analysis, … we removed 116,980 IOP measurements from 3,632 participants that occurred after prescription of IOP lowering medications to exclude values influenced by treatment."

We have also specified that the 356,987 IOP measurements from 69,756 individuals are <u>untreated</u> in the Abstract, Introduction, and Results sections.

3. **It may be helpful to present quantile-quantile plots and genomic inflation factor separately for each of the following collections: 60,032 non-Hispanic individuals of European descent, 5,790 individuals of Hispanic/Latino descent, 5,215 individuals of East Asian descent, and 2,070 individuals of African descent, to accompany the overall meta-analysis QQ plot currently shown in Supplementary Figure 1.**

As requested, we have now included in the Supplementary Figure 1 (b-e) the 4 QQ plots and corresponding genomic inflation factors for each of the 4 race/ethnicity groups (non-Hispanic white, Hispanic/Latinos, East Asian, and African American).

4. **Please include measures of heterogeneity for the meta-analysis (Phet, derived from Cochran's Q test and I2 index of heterogeneity) shown in Supplementary Table 1 and Supplementary Table 7.**

As suggested by the reviewer, we now provide measures of heterogeneity for the trans-ethnic meta-analysis results presented in Supplementary Table 1 and Supplementary Table 10 (originally Supplementary Table 7). We also added text in the Methods, as follows:

"Heterogeneity index, I^2 (0–100%) as well as *P*-value for Cochrane's Q statistic were assessed among groups."

5. **At first glance, many of the 47 loci overlap in terms of locus annotation with other eye diseases such as glaucoma and age-related macular degeneration. Checking through the list of loci and SNPs, could the authors provide linkage disequilibrium metrices (r2 and D') between their index SNPs and the SNPs previously reported to be associated with the other eye diseases?**
   For example, SNP rs28795989 at AFAP1 was reported in Table 2 by the authors to associate with IOP, whereas SNP AFAP1 rs4619890 was reported by Gharahkhani P et al., [Nature Genetics 2014] to be associated with increased POAG risk. In addition, SNP rs11732100, also at AFAP1, was reported to associate with increased POAG risk by Bailey JN et al., (reference #27 in the manuscript text). What is the relationship between the authors' top SNP rs28795989 with the other two SNPs (rs4619890 and rs11732100), for example? Filling in this detail would enhance the manuscript further by aiding the broad readership of the journal.

This is an excellent suggestion. We have now added a Supplementary Table 4 showing genetic associations at the IOP loci identified in the current study with other eye diseases (e.g. glaucoma, Axenfeld-Rieger syndrome, or Pitt-Hopkins syndrome) reported in previous studies. Supplementary Table 4 also provides the linkage disequilibrium metrices (R^2 and D') between our top SNPs and top SNPs from previous studies in European-Ancestry populations using a web-based bioinformatic tool (https://analysistools.nci.nih.gov/LDlink/) (Machiela MJ, Chanock SJ. Bioinformatics. 2015).

We have also added some text in the Results section to reflect this point, as follows:

""***Effect of the 47 IOP-associated loci on glaucoma***
… The two lead SNPs for *TMCO1* (rs4656461 and rs7518099) previously reported to be associated with POAG at genome-wide significance, were strongly correlated with our lead SNP rs6668108 in European-Ancestry populations (R^2 = 0.99 and 1.0, respectively) (Supplementary Table 4). The two lead SNPs for *AFAP1* (rs4619890 and rs11732100) previously reported to be associated with POAG at genome-wide significance, were moderately correlated with our lead SNP rs28795989 in European-Ancestry populations (R^2 = 0.25, and 0.51, respectively). Consistent with previous studies, we identified SNP rs2472493 as the lead SNP at the *ABCA1* locus. Our lead SNP rs9913911 in *GAS7* was relatively close (10.7 kb) to SNP rs9897123 previously reported, and was in linkage disequilibrium (LD) (R^2 = 0.54, D' = 0.95). We also found supportive evidence for the previously identified locus on *FOXC1* (P=2.9×10^{-4}) (Supplementary Table 3) with the same lead SNP rs2745572 reported by Bailey *et al*. (Supplementary Table 4)."

6. **Whilst scrutinizing Table 2, I note that SNP rs199800298 (ARHGEF12) could be linked to rs11827818, which was highlighted by Gharahkhani P et al., [Nature Genetics 2014]. This same marker has also been recently reported to associate with pseudo-exfoliation syndrome and glaucoma (Aung T et al., Nature Genetics 2017). As the authors emphasized a 'shared genetic etiology between glaucoma and related traits' in line 179 of the discussion section, some further analysis at this section could also aid in the overall relationship between genetic alleles and shared pleiotropy between the potentially related traits (e.g. pseudo-exfoliation syndrome is the commonest cause of POAG).**

In our Supplementary Table 4, in addition to glaucoma (POAG), we also summarized genetic associations at the IOP loci identified in the current study with other eye diseases (e.g. exfoliation syndrome, primary angle-closure glaucoma, or Axenfeld-Rieger syndrome) and related traits reported in previous studies. We have added a paragraph in the Results section to reflect this point, as follows:

"**Genetic association with related traits and eye diseases**
In addition to glaucoma risk, several loci for which the associations with IOP reached genome-wide significance were already known to influence the variation of ocular traits, or eye diseases (Supplementary Table 4). These findings extend previous studies showing overlapping GWAS regions between POAG and related traits. The current study identified a genome-wide significant signal with IOP at *EFEMP1* on chromosome 2. Common variants in *EFEMP1* (rs3791679 and rs1346786) have been shown to be associated with cup area, a specific optic disc measurement describing optic nerve morphology. Our lead SNP rs7426380 at *EFEMP1* was in LD with these two SNPs in European-Ancestry populations (R^2 = 0.33, D' = 0.98 for rs3791679, and R^2 = 0.49, D' = 0.98 for rs1346786) (Supplementary Table 4). Further, among our novel IOP loci identified which replicated, six have been previously associated with central corneal thickness, including *COL4A3*, *FAM46A-IBTK*, *ARID5B*, *FOXO1*, *SMAD3*, and *BANP-ZNF469*. Except for SNP rs1538138 at *FAM46A-IBTK*, all the lead SNPs at those loci previously associated with central corneal thickness were in high LD with our lead SNPs in European-Ancestry populations (R^2 ranged from 0.97 to 0.99, and D' ranged from 0.99 to 1.0) (Supplementary Table 4). We also identified a genome-wide significant signal with IOP on chromosome 9 at *GLIS3*, which is involved in the development of the eye, and has been recently identified as a new susceptibility locus for primary angle-closure glaucoma. Our lead SNP rs2224492 at *GLIS3* was correlated with the strongest SNP rs736893 reported by Khor *et al.* (R^2 = 0.71, D' = 0.85), and the two SNPs were relatively close (20.5 kb apart). These findings suggest a shared genetic etiology between IOP and related traits; further investigations could help to better understand their shared mechanisms and biological pathways.
Our study also identified novel IOP loci that have been previously involved in autosomal dominant Mendelian ocular conditions. Indeed, deleterious mutations in *FOXC1* and *PITX2* can cause Axenfeld-Rieger syndrome, an anterior segment dysgenesis disorder characterized by anomalies in the anterior chamber angle, including defects in the drainage structures of the eye. A major consequence of angle dysgenesis is an increase in IOP leading to the development of early-onset glaucoma, with *FOXC1* mutation carriers having a younger age at diagnosis in comparison to *PITX2* mutation carriers (6 vs. 18 years, respectively). Therefore, it is likely that common variants in *FOXC1* and *PITX2* mildly impact the drainage structures, and, in combination with defects of other genetic variants contribute to elevated IOP. Thus, it seems that there is a continuous spectrum of genetic predisposition to elevated IOP and related traits, from monogenic causative variants with high penetrance, to common polygenic variants with moderate to low penetrance."

7. **Supplementary Table 3 examined expression of the 47 genes or genes nearest to the index SNPs reported in this meta-analysis. Whilst this is helpful in the current manuscript, data in Table 2 showed that each locus was actually underlined by more than 1 gene, and sometimes, as many as 5-6 genes could be mapping to the 95% credible set underlined by the index SNP. In this light, could the authors**
   a) identify the total number of genes defined by the 95% credible set at each locus (this should be more than 47).
   b) annotate them for expression as per the Ocular Tissue Database and EyeSage, as the authors have done for the 47 closest genes.
   Doing so would be in keeping with Fritsche LG et al., (reference #81 in their manuscript text), which attempts to provide a more complete understanding of the genes and pathways implicated by the meta-analysis. Performing the DEPICT analysis (Supplementary Table 4) on a larger gene set implicated by the 95% credible set could uncover further biological insights implicated by the loci.

As suggested by the reviewer, we have now expanded the list of potential candidate genes in our IOP loci to 59 (instead of 47 genes or nearest genes, initially) using a Bayesian approach and the 95% credible set of variants. We provide all the credible sets of variants in a Supplementary Table (Supplementary Table 5), and we have added some text in the Methods section to describe the approach:

"In silico analyses

To produce the most thorough list of candidate genes within the 47 identified loci, we used a Bayesian approach (CAVIARBF) publicly available at https://bitbucket.org/Wenan/caviarbf. Briefly, for each of the 47 signals, we computed each variant's ability to explain the observed signal within a 2 Mb window (±1.0 Mb with respect to the original lead SNP) and derived, the smallest set of variants that included the causal variant with 95% probability (95% credible set). Previous studies have used similar approaches to prioritize variants near index SNPs for follow-up. These 47 credible sets included a total of 12,614 variants in 59 annotated genes (Supplementary Table 5)."

We then examined the expression of these additional genes in adult human eye tissues using the Ocular Tissue Database and EyeSAGE dataset, and reported the new results in Supplementary Table 6 and Supplementary Figure 2.

We would also like to clarify that DEPICT takes as input a set of independent, associated SNPs (and not genes) and automates all other steps (e.g. define positions in the human genome, create lists of genes at associated loci, etc.) (Pers TH *et al.* Nature Communications, 2015). For this reason, we ran DEPICT based on our independent genome-wide significant SNPs (all the 47 SNPs that achieved $P < 5x10^{-8}$ in the GERA GWAS). As a note, and according to DEPICT: "If no genes are within the locus defined by $r^2 > 0.5$, the gene nearest to the given lead SNP is included.".

Reviewer #2 (Remarks to the Author):

This manuscript reports a GWAS to search for factors that influence IOP in a remarkable analysis of 373,967 measurements from 73,2017 subjects identified through electronic medical record. A total of 47 new loci for IOP factors were identified, 40 of which were novel. This report makes a major contribution to the study of the genetic basis increasing the number of factors. The amount of IOP variability explained by known genetic factors was increased by 3-fold, but still accounts for a tiny fraction of the phenotype (3%).

This major analysis reports a valuable contribution by identifying many novel genetic factors that influence IOP.

Thank you to the reviewer for the positive feedback.

1. **I have one significant concern. The authors report identifying 47 genome-wide significant loci, 7 of which were previously reported. Of the 40 novel loci, only 14 met multiple measures threshold for significance in a second cohort (p = 0.05/40 = 0.0013). Suggestive p-values should not be evidence of confirmation. The authors should report throughout their report (title, abstract, results, discussion) that they discovered 14 novel loci (not 40). Replication is a key step in validating GWAS findings. The authors should clarify that the remaining 26 loci failed to replicate – i.e. they cannot confirm the association. It would be helpful to focus the discussion on the strongly replicated loci (i.e. FMNL2)**

   It is unclear why calculations for proportion of IOP variance; the DEPICT analyses; etc were calculated for loci / SNPs that did not replicate in a second cohort. What is the rationale for analyzing data that did not meet the threshold for statistical significance? Why not do this analysis with the confirmed loci?

Our rationale for presenting results for all loci is that in our point of view, presenting only the 14 loci that meet Bonferroni correction does not provide a full and accurate picture of the findings in this study. We identified 47 loci that reached genome-wide significance in our discovery sample, and we examined the lead SNPs in each of these loci for association in the published summary statistics of the largest previous GWAS study. We note that replication p-values are dependent in part on the size of the replication cohort, which in this case is smaller than our discovery cohort. Of our 47 lead SNPs, 7 were previously reported as genome-wide significant. Of the remaining 40 novel loci, there is a continuum of support for these associations. Of the 40 novel lead SNPs, 39 have associations in the same direction, 30 reach a nominal $p<0.05$ (compared to 2 expected by chance), and 14 are associated at a Bonferroni corrected significance level of $p<0.00125$ (versus 0.05 expected by chance). We believe that focusing on only the 14 loci that reached Bonferroni significance in our follow-up analyses would not accurately reflect the strength of our findings and would in effect throw away informative *in silico* findings for a large number of true associations.

However, we acknowledge the uncertainty and have altered the presentation of results. Further, we are removing emphasis on the 40 novel loci identified in our discovery sample throughout the manuscript.

We have changed the title to: "A large multi-ethnic genome-wide association study of intraocular pressure identifies <u>numerous</u> novel loci."

We have also made changes to the abstract and body of the manuscript to further detail the continuum of support that underlies our discovery findings, as follows:

In the Abstract:

"We identified 47 genome-wide significant IOP-associated loci ($P < 5 \times 10^{-8}$), of which 40 were novel. Most of them show at least nominal significance in the same direction in an external GWAS meta-analysis of 37,930 individuals of European and Asian descent."

In the Results section:

"**Replication in an independent external cohort**

We then tested the 40 lead single nucleotide polymorphisms (SNPs) representing each of the 40 independent loci for replication in an independent external meta-analysis consisting of 37,930 individuals of European and Asian descent from 19 studies. Of the 40 novel IOP-associated SNPs, 39 were associated with the same direction of effect in the replication sample, 14 replicated at Bonferroni significance ($P < 0.0013=0.05/40$), and an additional 16 were nominally significant ($P < 0.05$) (Table 2). Having a total of 75% of the SNPs meeting nominal significance is much more than the 5% expected by chance (28 vs. 2), suggesting most or all of these are also true associations."

We have also reorganized the Table 2 (Lead genome-wide significant SNP for each independent locus identified in the GERA discovery GWAS of IOP) to make a clear distinction between the 14 novel loci which replicated at Bonferroni significance, and the other loci, by stratifying the lead SNPs by the replication significance levels.

In the Discussion:

"In the large, ethnically diverse GERA cohort, we discovered 40 novel genome-wide significant IOP loci, and we provided additional support for their effect on IOP in an independent external meta-analysis."

We also provide additional support for these loci by adding information requested by reviewer 1, which includes adding tables on the association of these SNPs with other vision disorders in the literature and with glaucoma in our own sample.

2. **Page 10, PKHD1, PITX2 EFEMP1 were not confirmed as an IOP loci with the second cohort. The failure to replicate should be more clear in discussion of these (and other) loci. The discussion that milder mutations in genes that cause monogenic forms of glaucoma interesting, however, the specific examples are not well supported or described (the prior PlosOne report of a rare mutation in EFEMP1 in a single POAG family is suggestive but unconfirmed. The reference for PKHD1 is similarly suggestive).**

We agree with the reviewer that the two examples regarding a potential role of *PKHD1* and *EFEMP1* in monogenic forms of POAG were not well supported. Consequently, we have now deleted these two examples from the main text, as below:

"Our study also identified novel IOP loci that have been previously involved in autosomal dominant Mendelian ocular conditions. Indeed, ~~an exome sequencing study found a missense variant in *EFEMP1* that co-segregated with an autosomal dominant form of adult-onset POAG in an African-American family. Another exome sequencing study on a Chinese family with high frequency of POAG reported a non-synonymous mutation in *PKHD1*, suggesting that *PKHD1* is a susceptibility gene for POAG in the Chinese population. Our study extends these findings by identifying common variants at *EFEMP1* and *PKHD1*, which could contribute to glaucoma susceptibility through their influence on IOP. Similarly,~~ deleterious mutations in *FOXC1* and *PITX2* can cause Axenfeld-Rieger syndrome, an anterior segment dysgenesis disorder characterized by anomalies in the anterior chamber angle, including defects in the drainage structures of the eye. A major consequence of angle dysgenesis is an increase in IOP leading to the development of early-onset glaucoma, with *FOXC1* mutation carriers having a younger age at diagnosis in comparison to *PITX2* mutation carriers (6 vs. 18 years, respectively). Therefore, it is likely that common variants in *FOXC1* and *PITX2* mildly impact the drainage structures, and, in combination with defects of other genetic variants contribute to elevated IOP."

Other minor concerns

3. **Page 3- Line 52. The authors mention two features that might alter IOP variability within one of a patient's eyes. It would be helpful for them to begin by describing the known contributors to variability in IOP overall: time of day at which the measurement was taken (mentioned), corneal thickness, medications, and other demographic factors (age / race / etc). Also, the order at which measurements are made (between left and right eye) is a small, clinically insignificant difference, as their reference notes. Multiple different measurement techniques were employed (i.e. Goldmann tonometer, Tonopen XL, or others) and are another source of variability.**

This is an excellent suggestion. We now provide a more extensive description of the know contributors to variability in IOP overall in the Introduction, as following:

"IOP can vary due to different factors, including time of day at which the measurement was taken, corneal thickness, medications, different measurement techniques that were employed (i.e. Goldmann applanation tonometer, Tono-Pen XL, or others), and demographic factors such as age and racial/ethnic background. These sources of variability may reduce the power to detect IOP loci in studies that rely on a single, cross sectional measurement for each study participant."

4. **Page 5, line 84. It would be helpful to define lambda (genomic inflation)**

As suggested by the reviewer, we have now defined lambda as "genomic inflation factor" in the Results, as following:

"In our discovery GWAS analysis, we identified 47 independent genome-wide significant (P < 5x10-8) loci associated with IOP in the trans-ethnic meta-analysis (genomic inflation factor, λ of 1.12, reasonable for a sample of this size)."

5. **Page 10, line 189. Mutations in FOXC1 and PITX2 have been shown to cause autosomal dominant Axenfeld-Rieger Syndrome, which has abnormal iridocorneal angle structures as a key feature. It would be helpful to refer to these autosomal dominant (Mendelian conditions) as Axenfeld-Rieger Syndrome.**

As suggested by the reviewer, we have now specified "Axenfeld-Rieger syndrome" to refer to these autosomal dominant Mendelian ocular conditions caused by mutations in *FOXC1* and *PITX2*, as below:

"Our study also identified novel IOP loci that have been previously involved in autosomal dominant Mendelian ocular conditions. Indeed, deleterious mutations in *FOXC1* and *PITX2* can cause Axenfeld-Rieger syndrome, an anterior segment dysgenesis disorder characterized by anomalies in the anterior chamber angle, including defects in the drainage structures of the eye."

6. **Page 10, line 201-3. The authors should be more precise in their description of the anatomy of the outflow pathway – throughout the manuscript but here in particular. The trabecular meshwork is comprised of three layers (one of which is the juxtacanalicular layer, which is not separate from the TM as the sentence suggests).**

We have now modified the text in the Discussion, as follows:

"The meshwork is primarily composed of three layers, with the outermost region of the trabecular meshwork lined by the endothelial cells, forming the inner wall of Schlemm's canal (a modified capillary blood vessel that forms intra- and intercellular pores)."

7. **Page 15, 319. It would be helpful to readers (especially for online methods without space limitations) to provide the QC methods and outcomes for this large study rather than a reference.**

As suggested by the reviewer, we have now added details on QC procedures in the Methods section, as following:

"Genotype quality control (QC) procedures for the GERA samples were performed on an array-wise basis, as previously described. Briefly, we included SNPs with initial genotyping call rate ≥97%, allele frequency difference (≤0.15) between males and females for autosomal markers, and genotype concordance rate (>0.75) across duplicate samples. Around 94% of samples and more than 98% of genetic markers assayed passed QC procedures."

Again, we are grateful to both reviewers for pointing out these numerous additions and corrections, which have significantly enhanced our manuscript.

Reviewer #1 (Remarks to the Author):

The authors have been extremely responsive to the first round of reviews, and have revised the paper in great detail. I apologize for missing the following minor point in the first around, and thus request to be allowed to highlight this point as it would further help the reader.

a) Can the authors add the effect (or minor) allele frequencies for NHW, H/L, EAS, and AFR in Supplementary Table 1?

b) The relationship between polymorphisms mapping close to FNDC3B and keratoconus, CCT, IOP, and POAG have been reported a few times (e.g. Lu Y et al., [Stuart Macgregor & colleagues, Nat Genet 2013]), with slightly different SNPs showing association with the trait / disease. Looking at Supplementary Table 4, I am unsure why the R2 and D' matrices are labeled as NA between the index SNP rs7635832 and SNPs rs4894535, rs6445055, and rs4894796. As far as we know, these SNPs are polymorphic, and so should have r2 and D' matrices filled in. The same may apply to index SNP rs199800298 (an indel SNP) and rs11827818 and r58073046. The NA labels for monogenic rare mutations is of course appropriate.

Reviewer #2 (Remarks to the Author):

The authors have addressed my comments adequately, with one exception.

In the results section, "Replication in an independent external cohort", the authors last phrase, "suggesting that most or all of these are also true associations" overstates the significance of their finding. The authors are referring to a new analysis in this paragraph added to the revision, where they use $p < 0.05$ as a threshold for "nominal association". This analysis is concerning to me. It is not meaningful to note that several of the loci met a threshold for significance that is not appropriately corrected for multiple measures. The last phrase, "suggesting that most or all of these are true associations" is too strong. The reason for requiring replication and multiple measures corrections is to prevent false positives. These tenets should be adhered to by the authors and better reflected by their statements.

REVIEWERS' COMMENTS:

Reviewer #1 (Remarks to the Author):

The authors have been extremely responsive to the first round of reviews, and have revised the paper in great detail. I apologize for missing the following minor point in the first around, and thus request to be allowed to highlight this point as it would further help the reader.

a) Can the authors add the effect (or minor) allele frequencies for NHW, H/L, EAS, and AFR in Supplementary Table 1?

As requested, we have now included in the Supplementary Table 1 the effect allele frequencies for each of the 4 race/ethnicity groups (non-Hispanic white, Hispanic/Latinos, East Asian, and African American).

b) The relationship between polymorphisms mapping close to FNDC3B and keratoconus, CCT, IOP, and POAG have been reported a few times (e.g. Lu Y et al., [Stuart Macgregor & colleagues, Nat Genet 2013]), with slightly different SNPs showing association with the trait / disease. Looking at Supplementary Table 4, I am unsure why the R2 and D' matrices are labeled as NA between the index SNP rs7635832 and SNPs rs4894535, rs6445055, and rs4894796. As far as we know, these SNPs are polymorphic, and so should have r2 and D' matrices filled in. The same may apply to index SNP rs199800298 (an indel SNP) and rs11827818 and r58073046. The NA labels for monogenic rare mutations is of course appropriate.

For unknown reason, we obtained an error message when using the LDpair calculation tool (https://analysistools.nci.nih.gov/LDlink/) for the SNPs pairs at *FNDC3B* locus (SNP rs7635832 and SNPs rs4894535, rs6445055, and rs4894796). So, we have now estimated the linkage disequilibrium matrices (R^2 and D’) for those *FNDC3B* SNPs pairs using PLINK v1.9 (www.cog-genomics.org/plink/1.9/) software.

As a note, SNP rs199800298 (deletion on chromosome 11) at *ARHGEF12* locus was not in 1000 Genomes reference panel. So, we approximated the linkage disequilibrium matrices (R^2 and D’) between SNP rs199800298 and SNPs rs11827818 and r58073046 using our non-Hispanic white GERA sample.

We have added all of these LD values for SNPs pairs at *FNDC3B* and *ARHGEF12* loci in Supplementary Table 4.

Reviewer #2 (Remarks to the Author):

The authors have addressed my comments adequately, with one exception.

In the results section, “Replication in an independent external cohort”, the authors last phrase, “suggesting that most or all of these are also true associations” overstates the significance of their finding. The authors are referring to a new analysis in this paragraph added to the revision, where they use p< 0.05 as a threshold for “nominal association”. This analysis is concerning to me. It is not meaningful to note that several of the loci met a threshold for significance that is not appropriately corrected for multiple measures. The last phrase, “suggesting that most or all of these are true associations” is too strong. The reason for requiring replication and multiple measures corrections is to prevent false positives. These tenets should be adhered to by the authors and better reflected by their statements.

We have made changes to the abstract and body of the manuscript to further focus on the 14 IOP-associated loci that replicated at Bonferroni level of significance in the external replication cohort, as follows:

In the Abstract:

“We identify 47 genome-wide significant IOP-associated loci ($P < 5 \times 10^{-8}$); of the 40 novel loci, 14 replicate at Bonferroni significance in an external GWAS analysis of 37,930 individuals of European and Asian descent.”

In the Results section:

“**Replication in an independent external cohort**
Of the 40 novel IOP-associated SNPs, 39 were associated with the same direction of effect in the replication sample, and 14 replicated at Bonferroni significance ($P < 0.0013=0.05/40$) (Table 2).”

In the Discussion:

“In the large, ethnically diverse GERA cohort, we discovered 40 novel genome-wide significant IOP loci, of which 14 replicated at Bonferroni level of significance in an independent external meta-analysis.”
